# NIR‐Responsive injectable hydrogel cross-linked by homobifunctional PEG for photo-hyperthermia of melanoma, antibacterial wound healing, and preventing post-operative adhesion

**DOI:** 10.1016/j.mtbio.2024.101062

**Published:** 2024-04-24

**Authors:** Vahideh Nosrati-Siahmazgi, Samin Abbaszadeh, Kiyan Musaie, Mohammad Reza Eskandari, Saman Rezaei, Bo Xiao, Fatemeh Ghorbani-Bidkorpeh, Mohammad-Ali Shahbazi

**Affiliations:** a Department of Pharmaceutical Biomaterials, School of Pharmacy, Zanjan University of Medical Science, 45139-56184, Zanjan, Iran; b Department of Pharmacology, School of Medicine, Zanjan University of Medical Sciences, 45139-56111, Zanjan, Iran; c Department of Biomaterials and Biomedical Technology, University Medical Center Groningen, University of Groningen, Antonius Deusinglaan 1, 9713, AV Groningen, the Netherlands; d Department of Pharmacology and Toxicology, School of Pharmacy, Zanjan University of Medical Science, 45139-56184, Zanjan, Iran; e State Key Laboratory of Silkworm Genome Biology, College of Sericulture, Textile and Biomass Sciences, Southwest University, Chongqing, 400715, China; f Department of Pharmaceutics and Pharmaceutical Nanotechnology, School of Pharmacy, Shahid Beheshti University of Medical Sciences, Tehran, Iran

**Keywords:** Skin cancer, Photothermal therapy, Copper oxide nanoparticle, Wound repair, Abdominal adhesion

## Abstract

Current therapeutic approaches for skin cancer face significant challenges, including wound infection, delayed skin regeneration, and tumor recurrence. To overcome these challenges, an injectable adhesive near-infrared (NIR)-responsive hydrogel with time-dependent enhancement in viscosity is developed for combined melanoma therapy and antibacterial wound healing acceleration. The multifunctional hydrogel is prepared through the chemical crosslinking between poly(methyl vinyl ether-*alt*-maleic acid) and gelatin, followed by the incorporation of CuO nanosheets and allantoin. The synergistic inherent antibacterial potential of CuO nanosheets, the regenerative and smoothing effect of allantoin, the extracellular matrix-mimicking effect of gelatin, and the desirable swelling behavior of the hydrogel results in fast wound recovery after photothermal ablation of the tumor. Additionally, the hydrogel can serve as an alternative to sutures owing to its tissue adhesiveness ability, which can further render it the merits for accelerated repair of abdominal lesions while acting as a biocompatible barrier to prevent peritoneal adhesion.

## Graphical abstract

**Figure:**
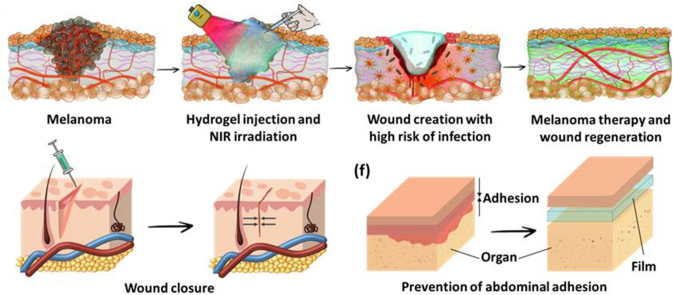

## 1 Introduction

Melanoma continues to be a daunting challenge for patients and healthcare professionals worldwide due to its aggressiveness and capacity for rapid metastasis, necessitating prompt intervention through the recognition of multifaceted challenges that impede the success of melanoma therapy [1]. Surgery followed by chemo/radiotherapy remains the typical treatment for melanoma; however, failure in complete eradication of tumor cells during the surgery, delayed repair of the damaged area, and toxicity of subsequent supplementary chemo/radiotherapy to the healthy tissues are still the main clinical problems to be addressed [2,3]. Moreover, the risk of post-operative bacterial infection deteriorates the condition through delayed wound regeneration [[4], [5], [6]]. Therefore, the combined eradication of tumor cells, induction of antibacterial effect, and tissue regeneration represent a promising approach for the current challenges of melanoma therapy [7,8].

Photothermal therapy (PTT) has made significant breakthroughs as a promising strategy for killing cancer cells via heat generation in the tumor site exposed to near-infrared (NIR) light [9]. Compared with chemo/radiotherapy, PTT is a minimally invasive strategy that can efficiently target cancer cells due to their nutrient deficiency and the hypoxic microenvironment compared to normal cells [[10], [11], [12]]. Among different metal oxide nanoparticles (NPs), copper oxide (CuO) has attracted significant attention as a photothermal agent due to its multifunctionality through inherent antimicrobial activity against gram-positive and gram-negative bacterial strains, which can be synergized by PTT to reduce the occurrence of post-treatment microbial infections in cancer ablation lesions. In addition, Cu ions released from the NPs can enhance the generation of vascular endothelial growth factor (VEGF), increment integrin expression, stabilize fibrinogen and collagen, and up-regulate copper-dependent enzymes crucial for tissue remodeling, fibroblast proliferation and re-epithelization [11,13,14]. Photothermal ablation of the tumor tissue is aimed to destroy the whole cancerous cells as reported in previous studies [[15], [16], [17]]. Therefore, angiogenesis effect and regenerative capacity of loaded components can promote the healing process in the next phase without any harmful effect on tumor re-growth since all the cancer cells are killed by PTT.

These remarkable benefits can be open up new avenues for proposing innovative combined cancer therapy and regeneration approaches using nanoparticle-incorporated hydrogels [1,[18], [19], [20]]. Among various multifunctional biomaterials, injectable hydrogel systems offer several advantages, including the delivery of therapeutic agents directly to the target site and enhancing their efficacy through sustained drug release, while minimizing off-target effects [2,4,7,21,22]. More importantly, adhesive hydrogels possess the unique ability to seal the tissue, facilitate wound closure, and regenerate tissue with less trauma and scarring compared with traditional sutures [[23], [24], [25], [26], [27]]. Meanwhile, the regenerative property of such hydrogels can be further expanded if their design allows them to act as a barrier to reduce abdominal adhesion in the case of intra-abdominal lesions caused by any type of cancer therapy in the abdominal region, surgery or trauma [28]. This is extremely important because intra-abdominal wounds lead to severe adhesions in up to 60 % of the patients, causing chronic pain, infertility, lethal intestinal obstruction, and the need for a second surgery [[29], [30], [31]]. Current strategies for the management of abdominal wounds and peritoneal adhesion show limited prevention and peritoneum repair [32]. Therefore, designing biodegradable hydrogels, which can combine wound healing with anti-adhesion effect would be beneficial.

**Fig. 1:**
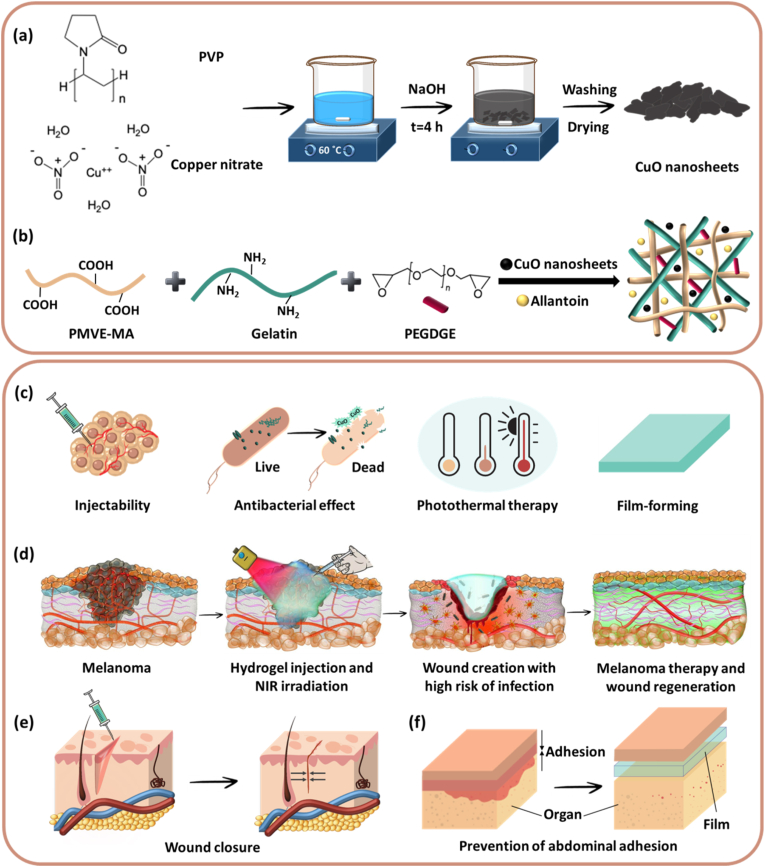
The schematic illustration of CuO nanosheets and hydrogel fabrication as well the applications of the developed multifunctional hydrogel. (a) CuO nanosheets were synthesized through the precipitation method. (b) The hydrogel was fabricated by the crosslinking reaction between PMVE-MA/gelatin and PEGDGE. CuO nanosheets and allantoin were also incorporated into the hydrogel network. (c,d) The injectable and film-formed hydrogel possesses anti-infection properties due to the intrinsic effect of CuO nanosheets synergized with hyperthermia via NIR light irradiation, which can be simultaneously applied for melanoma ablation and wound healing. The presence of CuO nanosheets and allantoin endows the hydrogel with the healing of wounds created in the tumor site after PTT. (e,f) The hydrogel can be utilized as a promising alternative to suturing and as an anti-adhesion candidate for postsurgical wounds.

Inspired by the facts above, we designed a multifunctional hydrogel consisting of poly(methyl vinyl ether-*alt*-maleic acid) (PMVE-MA) and gelatin through the ring-opening of epoxide groups of poly(ethylene glycol)diglycidyl ether (PEGDGE) and bridging between carboxylic acid groups of PMVE-MA and amines in gelatin. The viscosity of the formed hydrogel increases over time, enabling injection at the first hours of formation, followed by creating a strong gel in the target site. PMVE-MA as a hydrophilic, biocompatible, and bioadhesive polymer, forms hydrogels with a large amount of exudate absorbance at the wound site, which is an essential criterion for an ideal wound healing formulation. Gelatin as a natural polymer, possesses desirable properties, including biodegradability, ECM mimicking, cell attachments, proliferation, and remodeling. Additionally, the incorporation of allantoin within the hydrogel could synergistically accelerate the wound healing process by reducing skin irritation and providing keratolytic and anti-inflammatory activity [33,34]. As shown in the Fig. 1, the final CuO nanosheet-incorporated hydrogel possesses injectability, adhesiveness, NIR-responsiveness, anti-tumor effect, antibacterial property, and anti-adhesion characteristic, which make it an ideal platform for NIR-based cancer therapy, wound closure, and healing of wounds in different part of the body.

## 2 Results and discussion

### 2.1 Fabrication and characterization of CuO NPs

**Fig. 2:**
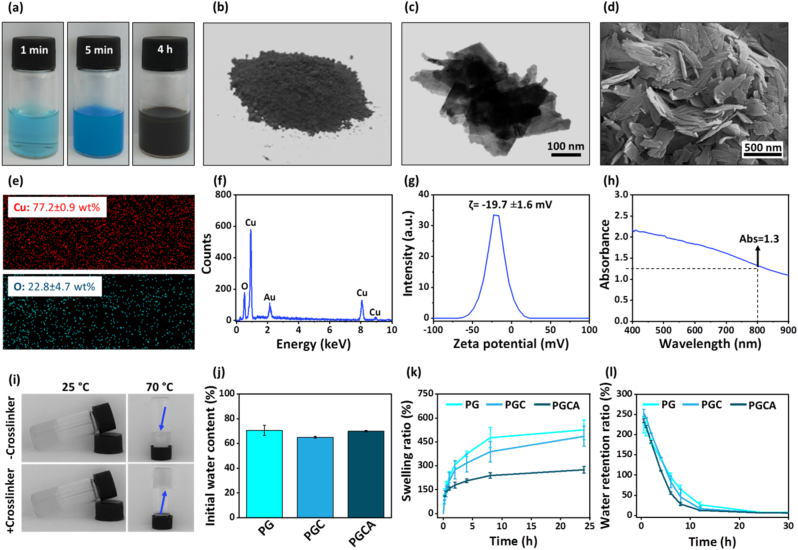
Physicochemical characterization of CuO nanosheets and hydrogels. (a) Representative images of CuO nanosheet formation over 4 h. (b) Synthesized powder of the CuO nanosheets. (c,d) TEM and FE-SEM images of CuO nanosheets. For better understanding of the shape of the CuO, more TEM images are presented in Fig. S1. (e,f) The elemental mappings of Cu and O elements and EDAX spectrum of CuO nanosheets. (g) The Zeta-potential of CuO nanosheets. (h) UV–Vis spectra of CuO nanosheets. (i) The inversion tube test of the PGCA hydrogel with and without chemical crosslinker at room temperature and 70 °C. (j) Initial water content, (k) swelling ratio at 37 °C and pH 7.4, and (l) water retention capacity of the PG, PGC, and PGCA hydrogels. Data are reported as mean ± standard deviation (SD; N = 3).

The CuO NPs were successfully synthesized through the precipitation method and the color changed from light blue to dark blue after NaOH addition, and subsequently, to dark brown after 4 h (Fig. 2a). The obtained CuO powder can be observed in Fig. 2b. The transmission electron microscopy (TEM) image of CuO NPs indicated a sheet-like morphology with lengths ranging from 100 to 400 nm (Fig. 2c). Moreover, the field-emission scanning electron microscopy (FE-SEM) image confirmed the sheet-like morphology of CuO NPs (Fig. 2d). The elemental compositions of synthesized CuO nanosheets were verified by the energy-dispersive X-ray (EDAX) analysis (Fig. 2e and f). The weight percentages of Cu and O were 77.2 ± 0.9 and 22.8 ± 4.7, respectively. Also, the spectrum confirmed the presence of Cu and O in high percentages without any impurity. Notably, the gold (Au) peak is due to the coating of the sample with it before FE-SEM imaging. The zeta potential (ζ) value for CuO nanosheets was −19.7 ± 1.6 mV (Fig. 2g) due to the abundant negatively ionized oxygen groups of the nanosheets, which causes an electrostatic repulsion to keep them stable and prevents their aggregation by overcoming the Van Der Waalsʼ attraction force. The UV–Vis spectra of CuO nanosheets with a concentration of 400 μg/ml in deionized water (DI) showed a good light absorption capacity at the wavelength of 808 nm as a consequence of the d-d electronic transition of Cu ions (Fig. 2h). This feature proposes CuO nanosheets as a possible candidate for PTT, which can efficiently absorb NIR light.

### 2.2 Fabrication and characterization of hydrogels

The PG hydrogel network was formed through the reaction between the two ring-opened epoxide groups of PEGDGE with carboxylic acid and amine groups of PMVE-MA and gelatin, respectively. CuO nanosheets alone or with allantoin were added into the PMVE-MA solution to acquire PGC and PGCA hydrogels, respectively, followed by combining with the crosslinker and gelatin solution to form the hydrogel. The detailed methodology is explained in section 1.2 of the Supplementary Information.

The tube inversion test of the PGCA hydrogel with and without crosslinker (Fig. 2i) showed that the hydrogel with and without crosslinker has no fluency at 25 °C 3 h after preparation. The samples were then placed in a water bath at 70 °C, showing the one without the crosslinker has high fluidity. This confirmed that there was no interaction between gelatin and PMVEMA in the absence of the crosslinker and gelation at room temperature was due to the intrinsic solidification of the gelatin at temperatures lower than ca. 40 °C. On the other hand, the structure of the hydrogel was well-formed and did not flow under heat treatment, confirming the successful chemical bonding of the polymers within the backbone of the hydrogel. We also observed that CuO nanosheets does not have any effect on the gelation time of the hydrogel because the gel formation occurs through the ring-opening of epoxide groups of poly(ethylene glycol)diglycidyl ether (PEGDGE) and its crosslinking with carboxylic acid groups of PMVE-MA and amines in gelatin. The CuO does not have any amine or COOH functionality on its surface. Therefore, it does not interfere with the gel formation through reaction with the crosslinker. That is the main reason for observing no change in the gelation time after addition of the CuO nanosheets.

The initial water content evaluation showed the high water content of PG, PGC, and PGCA hydrogels, and no significant difference was observed among the groups (Fig. 2j). The swelling rate for all hydrogels was rapid at the first 8 h of the study and then reached to equilibrium because of the limited expandability potential of the hydrogel network. PGC hydrogel showed a slightly lower swelling ratio in comparison to PG hydrogel, due to the incorporation of CuO nanosheets within the hydrogel network. PGCA hydrogel showed the lowest swelling ratio owing to the interaction of allantoin with the polymeric backbone of the hydrogel and stronger bondings that limit water penetration (Fig. 2k). However, the obtained swelling ratio is desirable enough to absorb tissue exudates, maintain a moist wound environment, and also provide a microenvironment for cell migration and proliferation for regenerative applications.

The water retention capacity of different hydrogels was also carried out as another important criterion for wound regeneration since adequate moist content can accelerate the re-epithelialization process [35] and allow the removal of the hydrogel without adhesion and pain.

The water retention ratio of all hydrogels followed a similar trend with negligible differences (Fig. 2l). TEM images of CuO nanosheets (Fig. S1), SEM images of PGCA hydrogel (Fig. S2), yield, and degradation of the hydrogels (Figs. S3a and b) are available in the Supplementary Information. Also, the results and discussions of other physiochemical characterizations, including attenuated total reflection-Fourier transform infrared (ATR-FTIR; Fig. S4), **X-ray diffraction (**XRD; Fig. S5), thermogravimetric analysis (TGA; Fig. S6), and derivative thermogravimetry (DTG; Fig. S6) are available in the Supplementary Information, demonstrating the successful fabrication of the CuO nanosheets and the hydrogel. The advantages of the developed hydrogel include facile fabrication without using any toxic crosslinker or organic solvent, controllable gelation, injectability, high porosity, tunable stiffness, and ease of drug/nanoparticle loading into it without affecting its physicochemical properties.

### 2.3 Rheological and mechanical properties of the hydrogels

**Fig. 3:**
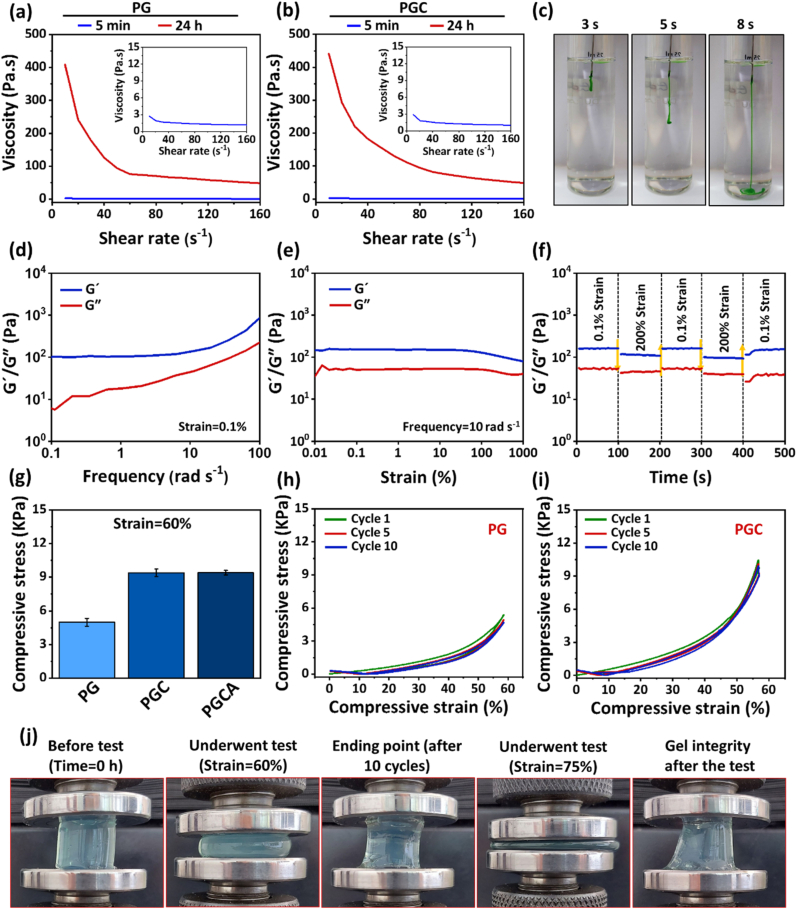
Rheological and mechanical characteristics of hydrogels. Viscosity measurement of (a) PG and (b) PGC hydrogels. (c) The injectability representation of colored PGC hydrogel at 3, 5, and 8 s using a 21-G needle. (d) Frequency sweep test at the strain of 0.1 %, (e) strain amplitude sweep test at the frequency of 10 rad s^−1^, and (f) alternate step strain test of PGC hydrogel 48 h after preparation between strains of 0.1 and 200 %. (g) The compressive stress of PG, PGC, and PGCA hydrogels at the strain of 60 %. The compressive test of the (h) PG and (i) PGC hydrogels after 10 loading-unloading cycles at strains of 0–60 % on day 10 after preparation. (j) The representative photographs of PGCA hydrogel before, during, and after the compression test.

The smooth injectability is a desirable feature for developing novel hydrogels, which can be characterized through the study of rheological features. The viscosity of the hydrogels as a function of time and the shear rate was evaluated. It is shown that the viscosity increases over time for the hydrogel due to the time-dependent crosslinking of the polymeric chains, while the elevation of the shear rate reduces the viscosity (Fig. 3a and b). This observation verifies the shear-thinning capacity of the hydrogels, which is an important criterion for an injectable hydrogel (Fig. S7a). It is noteworthy to mention that PGC hydrogel showed higher viscosity in comparison to PG hydrogel due to the incorporation of CuO nanosheets, which could enhance the strength of hydrogel. Moreover, injectability was assessed by monitoring the force required for the injection of PGCA hydrogel 2 and 4 h post-fabrication at room temperature (Fig. S7b). The results showed an initial upward gradient overcoming syringe friction, followed by a plateau as the hydrogel exited the needle. A higher force was needed at 4 h due to the accelerated crosslinking reaction, which is in accordance with viscosity data.

In addition, the injectability of the hydrogel in the PBS buffer (pH 7.4) using a 10-ml syringe with a 21-gauge (G) needle confirmed the smooth ejection of the hydrogel without any obstruction and discontinuation (Fig. 3c and Video S1). Therefore, the fabricated hydrogel can be injected at the first hours of fabrication, and then viscosity will be increased due to the enhanced chemical crosslinking over time to enhance the retention time of the hydrogel at the injection site for cyclic PTT.

The viscoelastic properties of the hydrogels were further studied at 48 h and 10 days after preparation. As shown in Fig. 3d and Fig. S8a, the G′ (storage modulus) value of PG and PGC hydrogels were larger than G″ (loss modulus) value at the frequency range of 0.1–100 rad s^−1^, indicating hydrogel stability over this range without any intersection. The G′ and G″ measurements on day 10 showed an increase for both hydrogels in comparison to 48 h, confirming the viscosity enhancement over time (Figs. S9a and S9d). Also, the incorporation of CuO nanosheets in PGC hydrogel caused an increment of G′ and G″ values compared to the PG hydrogel.

As shown in Fig. 3e, S8b, S9b, and S9e, G′ was bigger than the G″ value for all hydrogels in the tested strain range of 0.01–1000 %, indicating good mechanical strength 48 h and 10 days after hydrogels preparation. The results showed that the gel networks were not broken even under high-magnitude strains, which is suitable for placement on the wound bed without any concern related to rupture in the structure of the hydrogel.

Alternate step strain measurements showed a slight change in the G′ and G″ values of PG and PGC hydrogels in five continuous step strains fluctuated between 0.1 % and 200 % at 48 h (Fig. 3f and Fig. S8c) and 10 days (Figs. S9c and S9f) after preparation, indicating the stability of the hydrogel. Also, the slight decrease of the G′ and G″ values at the strain of 200 % was desirably recovered very quickly in all cycles, which demonstrates high mechanical property of the hydrogel. The high mechanical stability of the hydrogels is attributed to the chemical crosslinks, hydrogen bonds, and electrostatic interactions that facilitate the high interaction of polymer chains [36].

Compressive stress-strain measurement was also conducted to evaluate the mechanical behavior of the hydrogels after 10 loading-unloading cycles of 0–60 % compressive strain without any relaxation time. The compressive stresses of hydrogels at 60 % compressive strain were 4.99, 9.39, and 9.40 KPa for PG, PGC, and PGCA (Fig. 3g), respectively, indicating the role of CuO nanosheets in enhancing the mechanical property of the hydrogel. In addition, none of the hydrogels revealed an obvious decrease in compressive stress after 10 cycles, indicating their stability under mechanical forces (Fig. 3h,i, and Fig. S10). Moreover, after withstanding 60 % strain for 10 cycles, the hydrogels tolerated 75 % of compressive strain and were able to quickly reverse to the original shape with a very slight deformation after removal of the loading force (Fig. 3j and Fig. S11). Hence, the results indicated that PGC and PGCA hydrogels had adequate resilience, toughness, and tolerance to compressive strain without significant deformation [37].

### 2.4 Photothermal performance of the CuO nanosheet and hydrogels

**Fig. 4:**
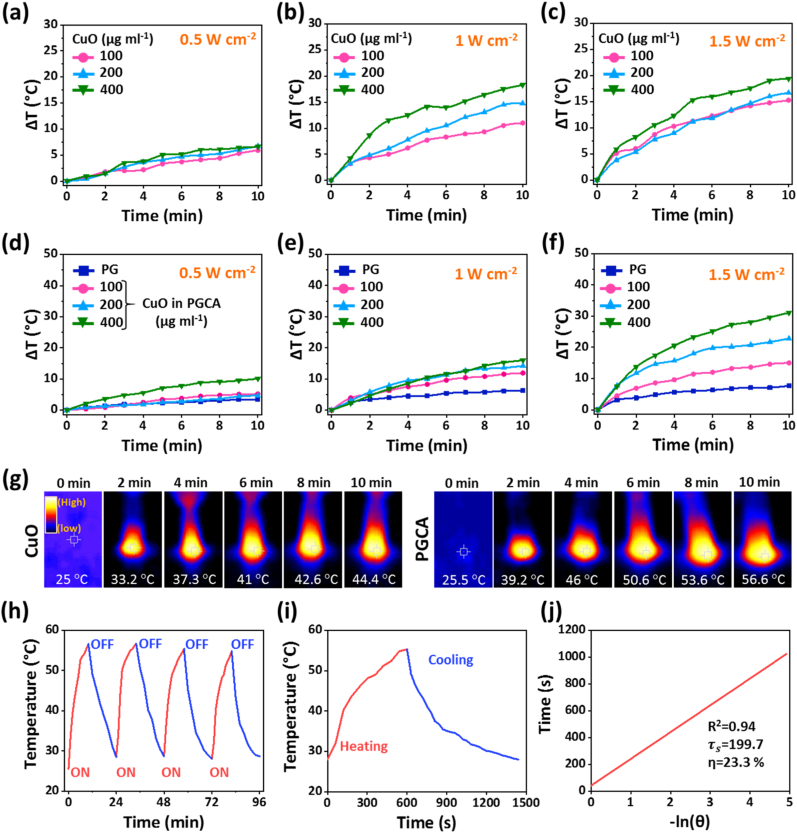
Thermal plots of (a–c) CuO nanosheets as well as (d–f) PG and PGCA hydrogels with different concentrations of CuO nanosheets (100, 200, and 400 μg ml^−1^) under NIR irradiation (808 nm) with power densities of 0.5, 1, and 1.5 W cm^−2^. (g) Infrared thermal photographs of CuO nanosheets and PGCA hydrogel containing 400 μg ml^−1^ of nanosheets under 10 min laser irradiation (808 nm) with a power density of 1.5 W cm^−2^. (h) Photothermal stability of the PGCA hydrogel under four repeated ON/OFF cycles of NIR light irradiation (808 nm, 1.5 W cm^−2^). (i) The photothermal plot of PGCA hydrogel under NIR irradiation with a power density of 1.5 W cm^−2^ for 10 min, followed by 14 min of cooling stage. (j) The curve of cooling time versus the negative natural logarithm of the temperature obtained from the cooling state of Fig. 4i.

To determine the photothermal effect, CuO nanosheets (100, 200, and 400 μg ml^−1^), PG hydrogel, and PGCA hydrogel (containing 100, 200, and 400 μg ml^−1^ of CuO nanosheets) were exposed to NIR laser irradiation (808 nm) with 0.5, 1, and 1.5 W cm^−2^ power density for 10 min. CuO nanosheets showed a concentration-dependent increment of the temperature with different power densities (Fig. 4a–c). The same trend was observed for the PGCA hydrogel containing 100, 200, and 400 μg ml^−1^ of CuO nanosheets (Fig. 4d–f), which demonstrated a temperature rise of 11.9, 14.2, and 16.0 °C with a power density of 1 W cm^−2^, and 15.0, 22.8, and 31.1 °C with a power density of 1.5 W cm^−2^**.** However, the PG hydrogel showed a very slight increase in temperature with the different power densities. Moreover, the PGCA hydrogel showed a higher temperature increment compared to CuO nanosheets at the same concentrations using different power densities. The thermal images of CuO nanosheets and PGCA hydrogel containing 400 μg ml^−1^ of nanosheets under laser irradiation with 1.5 W cm^−2^ are shown in Fig. 4g. The concentration of 400 μg ml^−1^ of nanosheets and the power density of 1.5 W cm^−2^ caused temperature elevation to 56.6 °**C** for the PGCA hydrogel under 10 min of laser irradiation, which was chosen as an optimum condition for cancer ablation that can lead to cancer cell death through necrosis and apoptosis [38]. Additionally, the PGCA hydrogel showed approximately the same initial and final temperatures at four ON/OFF cycles of the laser irradiation (1.5 W cm^−2^, 10 min), indicating the excellent photothermal stability of the nanosheets, which enables multiple exposure of NIR irradiation and heat generation after single injection of the hydrogel into the melanoma cancer site (Fig. 4h). To evaluate the photothermal conversion efficiency (η), the NIR laser (1.5 W cm^−2^, 10 min) was irradiated to the PGCA hydrogel (1 ml) and then the temperature was recorded at the cooling stage (Fig. 4i). Subsequently, the curve of cooling time (t) versus the negative natural logarithm of the temperature driving force (−ln θ) was obtained (Fig. 4j) [39]. The obtained η value was calculated to be 23.3 % according to Equation (8) described in the experimental section of the Supplementary Information.

### 2.5 Hemocompatibility and cellular viability

**Fig. 5:**
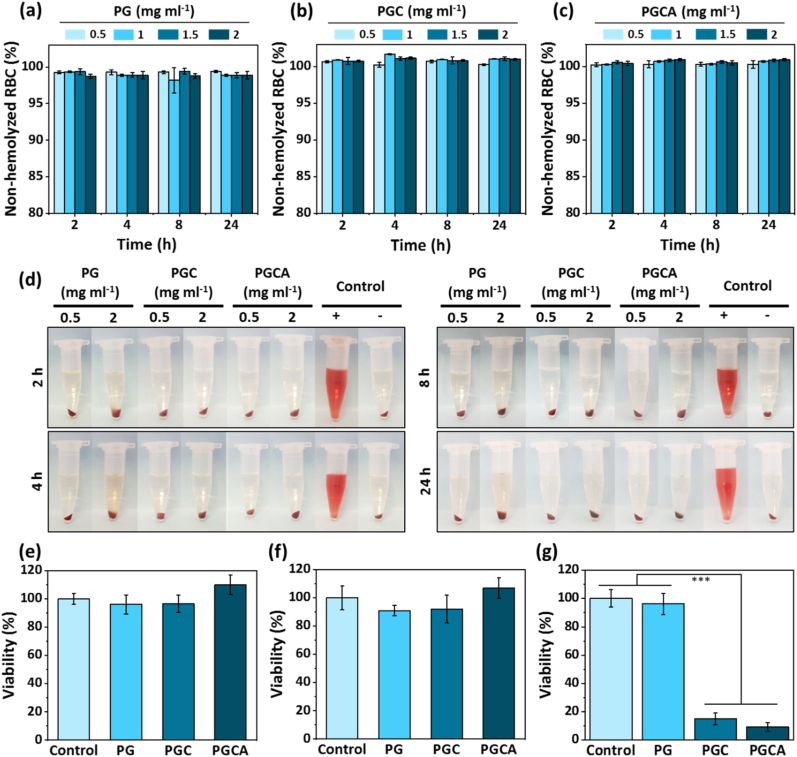
In vitro hemotoxicity of the (a) PG, (b) PGC, and (c) PGCA hydrogels (0.5, 1, 1.5, and 2 mg ml^−1^) after 2, 4, 8, and 24 h incubation with human RBC at the room temperature. (d) The representative images of the hemolytic activity of various hydrogels with 0.5 and 2 mg ml^−1^ and control groups after 2, 4, 8, and 24 h incubation with human RBC. Data are reported as mean ± SD (N = 3). The viability of B16F10 cancer cells after treatment with PG, PGC, and PGCA hydrogels at 37 °C for (e) 24 h and (f) 48 h. (g) The in vitro photothermal killing effect of PG, PGC, and PGCA hydrogels was evaluated under NIR laser irradiation (808 nm, 1.0 W cm^−2^ for 10 min). The PGC and PGCA results were significantly different from the control and PG groups. Data are reported as mean ± SD (N = 4), (***p < 0.001).

To evaluate the hemocompatibility of PG, PGC, and PGCA hydrogels, the hemolysis ratio was studied after being exposed to human red blood cells (RBCs) for 2, 4, 8, and 24 h in different concentrations. The quantitative data showed that the hemolysis ratio of all three hydrogels were below 1 % (Fig. 5a–c), indicating excellent hemocompatibility of the hydrogels based on the international level of allowed hemolysis (5 %) for biomaterials [40]. Moreover as shown in Fig. 5d, the supernatant of all hydrogels showed a transparency similar to the negative control group (PBS, pH 7.4) after centrifugation, while it was bright red for the positive control group (DI). The results indicated the desirable hemocompatibility of the prepared hydrogel for biomedical applications.

Moreover, the safety of hydrogels was assessed on the B16F10 cancer cells 24 and 48 h after incubation with the cells. As shown in Fig. 5e and f, all three hydrogels demonstrated high safety with cell viability of higher than 90 % at two different time points for all of them. The cell viability of PGCA hydrogel was higher than 100 %, which can be attributed to the proliferative activity of allantoin. The viability of NIH/3T3 and HDFa normal fibroblasts after treatment with PG, PGC, and PGCA hydrogels also demonstrated high safety (Figs. S13a and b). These findings suggest that the hydrogel is biocompatible and can be used for effective wound healing.

The in vitro anticancer effects of hydrogels were assessed on B16F10 cells through NIR-mediated heat generation. As shown in Fig. 5g, PGC and PGCA hydrogel killed more than 85 % and 90 % of cancer cells, respectively, owing to induced hyperthermia. These results are in line with the studies that reported temperatures above 46 °C lead to cancer cell necrosis and apoptosis [41,42].

### 2.6 In vivo toxicity of the hydrogels

**Fig. 6:**
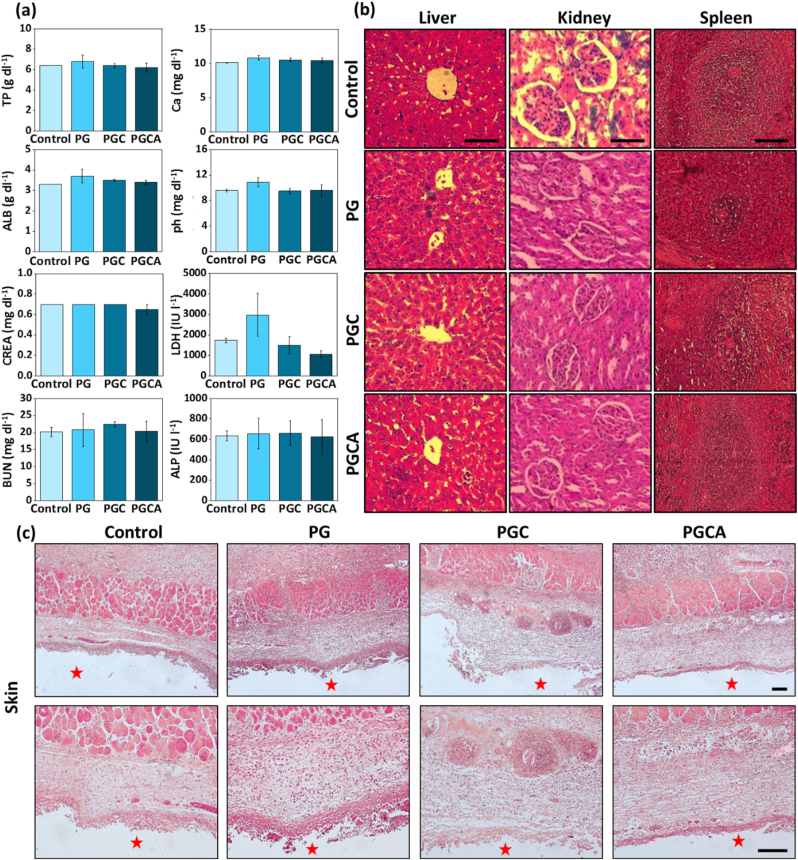
In vivo toxicity evaluation of PG, PGC, and PGCA hydrogels. (a) Biochemical factors of different treated groups after 14 days. Data are reported as mean ± SD (N = 4). The statistical analysis was performed by One-way ANOVA (*p < 0.05 *vs*. control group). (b) Hematoxylin-eosin (H&E) staining of liver, kidney, spleen, and (c) skin tissues 14 days after injection of the hydrogels into the dorsal side of the rat. (Star symbols represent the injection site of various hydrogels). Scale bar = 100 μm.

The in vivo toxicity was determined by subcutaneous injection of the PG, PGC, and PGCA hydrogels (500 μl) into the dorsal side of the rat, followed by checking the histology of different organs as well as biological and blood parameters after 14 days. No macroscopic infection and necrosis were observed at the injection site of the hydrogels during the study. As shown in Fig. 6a there was no significant change in all biochemical factors compared with the control group, including total protein (TP), calcium (Ca), albumin (ALB), phosphor (Ph), creatinine (CREA), lactate dehydrogenase (LDH), blood urea nitrogen (BUN), and alkaline phosphatase (ALP). Also, no considerable change was observed in hematological factors in comparison to the control group, including the blood level RBCs, hemoglobin (HGB), hematocrit (HCT), platelet (PLT), white blood cells (WBCs), neutrophils (NEUT), lymphocytes (LYMPH), and monocytes (MONO) (Fig. S14). Moreover, no significant histopathological lesions and structural abnormalities or necrosis were observed in the hydrogel-treated groups (Fig. 6b and Fig. S15). The analysis of liver tissue showed lower infiltration of inflammatory cells in the PGCA hydrogel-treated group compared to PG and PGC hydrogel, owing to the anti-inflammatory effects of allantoin [43,44]. In addition, micrographs of the kidney and spleen showed normal renal glomerulus and tubes without any necrosis as well as the normal shape of the spleen sinus. The skin tissue at the injection site demonstrated a moderate accumulation of inflammatory cells in the PGC hydrogel-treated group, due to the toxicity of CuO nanosheets, which decreased in PGCA hydrogel, owing to the presence of allantoin (Fig. 6c). In the body, Cu homeostasis is tightly maintained through a complex system of Cu transporters and chaperone proteins. This intricate system guarantees the delivery of Cu to essential proteins without causing cellular damage. Disruption in Cu hemostasis can lead to an overabundance of Cu, resulting in possible toxicity. When CuO nanosheets are introduced into a biological system (e.g., human body), they might be subject to processes such as phagocytosis, cellular uptake, or potential biotransformations within cells and affect metabolic pathways within living organisms. Some studies have shown that CuO nanoparticles can induce oxidative stress, disrupt cellular signaling pathways, and influence cellular metabolism at high concentrations [[45], [46], [47]]. In our study, based on the in vivo toxicity results, which were in line with in vitro data, PGCA hydrogel is proposed as a safe therapeutic material for biomedical applications in the tested concentration. Also, the in vivo degradation study showed no residual hydrogel at the injection site after 14 days.

### 2.7 Antibacterial activity of hydrogels in vitro/in vivo

**Fig. 7:**
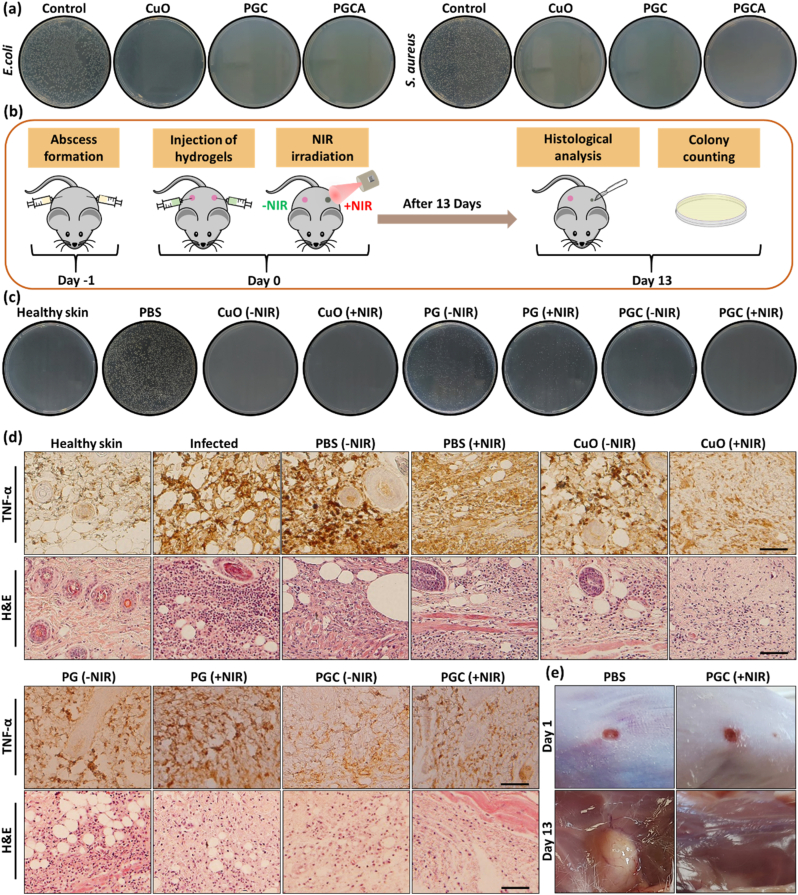
(a) Photographs of in vitro antibacterial performance of CuO nanosheets, PGC, and PGCA hydrogels. (b) The schematic illustration of in vivo antibacterial assay. (c) Photographs of MRSA bacterial colonies in healthy skin, infected skin, PBS, CuO nanosheets, PG, and PGC-treated groups with or without NIR laser irradiation. **(**d) The images of histological analysis and TNF-α expression in the infected skin tissues after 13 days. (e) Macroscopic images of subcutaneous abscess on days 1 and 13 for PBS and PGC (+NIR)-treated mice. Scale bar = 100 μm.

The infection caused by the immune deficiency in cancer patients can lead to the secretion of inflammatory factors which develops cancer metastasis and skin ulcers [48,49]. To overcome these obstacles, hydrogels with antibacterial abilities are promising candidates for biomedical applications [50]. To evaluate the antibacterial effect of nanosheets and hydrogels against *Staphylococcus aureus* (*S. aureus*; a gram-positive bacteria) and *Escherichia coli* (*E. coli*; a gram-negative bacteria) the in vitro colony‐counting test was used. As shown in Fig. 7a, no colony growth was observed in CuO nanosheets, PGC, and PGCA hydrogel-treated groups (>99 % antibacterial activity), due to the effect of CuO nanosheets on the generation of reactive oxygen species and adherence of nanosheets to the bacterial cell membrane, which leads to enhanced membrane permeabilization and cell lysis as well as DNA and protein damage [51,52]. Moreover, the antibacterial activity of maleic acid in the structure of the PMVE-MA copolymers assisted in killing bacteria [53].

The in vivo antibacterial activity of CuO nanosheets, PG, and PGC hydrogels with and without NIR irradiation (808 nm; 1.5 W cm^−2^) was also evaluated by murine subcutaneous abscess model using **methicillin-resistant *S. aureus* (**MRSA) bacteria. The abscesses formed 24 h after the injection, followed by treating the mice with a subcutaneous injection of 100 μl of PBS, PG, and PGC at the infection site (Fig. 7b). After laser irradiation, the local temperature in CuO (+NIR) and PGC (+NIR) increased to 52 °C within 4 min and adjusted at this temperature for 2 min, which is an efficient temperature for killing bacteria. However, the temperature for the other groups had a mild increase to about 40–42 °C in 6 min. On day 13, to determine the bacterial growth, the swab samples were taken from the infected tissue and cultured ex vivo using the standard plate counting method. CuO nanosheets (±NIR) and PGC (+NIR) groups showed no bacterial growth (>99 % antibacterial activity). PGC (-NIR)-treated group displayed very low amounts of bacteria (Fig. 7c), which is attributed to the inherent antibacterial activity of CuO nanosheets. PG (±NIR) groups demonstrated significantly lower bacteria growth than the PBS-treated group, due to the antibacterial activity of PMVE-MA. In addition the mild increased temperature in PG (+NIR) group resulted in a higher killing effect in comparison to PG (-NIR). Moreover, the healthy skin group, as the negative control, showed no bacterial growth. Furthermore, histological analysis with two different magnifications (Fig. 7d and Fig. S16) demonstrated the least inflammatory exudates and infiltration of immune cells in the PGC (+NIR) group owing to the antibacterial effect of CuO nanosheets. Also, PG (-NIR) and PGC (-NIR)-treated groups showed less inflammation and neutrophil infiltration in comparison to CuO (-NIR) due to the anti-inflammatory effect of gelatin. However, high neutrophil infiltration and expression of TNF-α were observed in the infected and PBS groups, confirming the severe infection caused by MRSA. Moreover, the macroscopic image of the infection site (Fig. 7e) depicted a significant amount of pus for the PBS group, while no pus was noticed for the mice treated with PGC (+NIR) at the end of the experiment. These results suggested the hydrogels containing CuO nanosheets under laser irradiation are an excellent antibacterial platform against *E. coli*, *S. aureus*, and MRSA bacteria.

### 2.8 In vivo antitumor effect

**Fig. 8:**
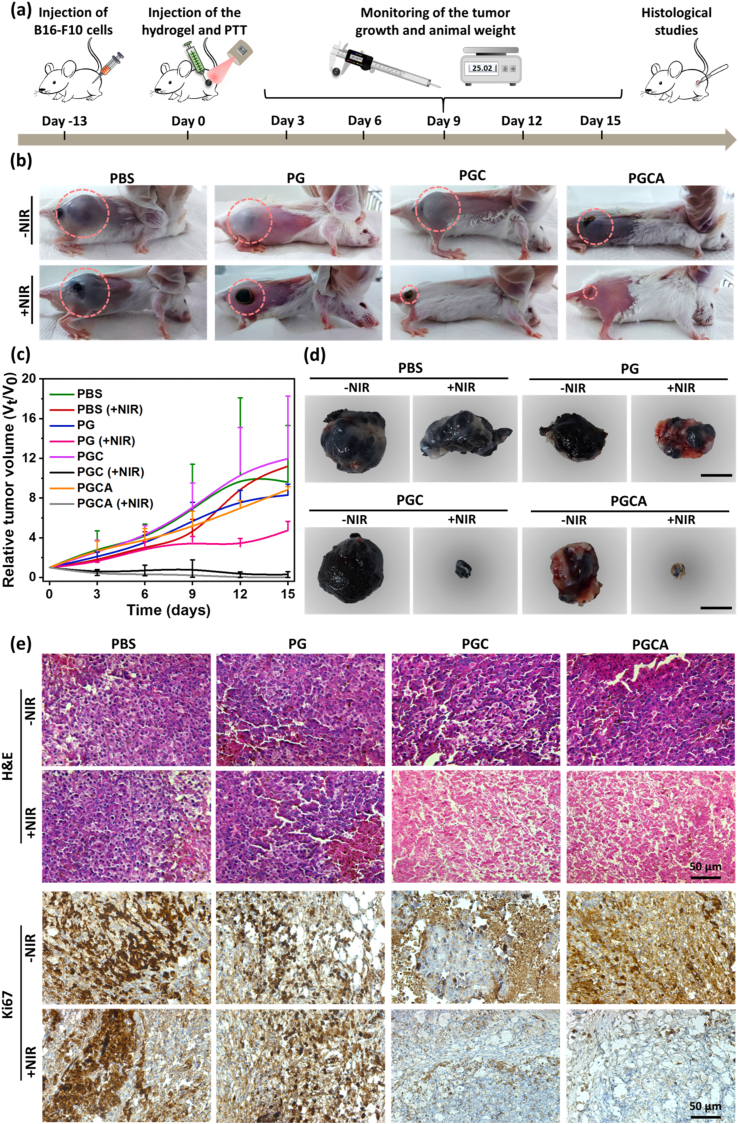
(a) Schematic illustration of in vivo PTT-based anti-cancer therapy. (b) Representative photographs of mice treated with PBS (±NIR), PG (±NIR), PGC (±NIR), and PGCA (±NIR) on day 15. (c) Relative tumor volume of different treated groups. (d) The representative digital images of tumors after different treatments with and without NIR irradiation on day 15. Scale bar = 1 cm. (e) H&E and Ki67 staining of the tumor tissues 15 days after treatment. Higher magnification of H&E images are presented in Fig. S18b for better demonstration of necrosis and nuclear damage of tumor tissue. Results are presented as mean ± SD (N = 6). Scale bar = 50 μm.

The antitumor assessment was conducted in B16–F10 bearing female BALB/c mice after subcutaneous injection of PBS, PG, PGC, and PGCA with and without NIR irradiation (Fig. 8a). Following laser irradiation, the temperature of the tumor site in PGC and PGCA-treated mice rapidly reached over 52 °C within 2.5 min and was controlled manually not to exceed a higher temperature and remained stable for 4 min. The other groups did not show a significant temperature rise after laser irradiation for 6.5 min. The photographs of treated mice with different formulations on day 15 with and without NIR irradiation are shown in Fig. 8b. The PBS group had a more than 10-fold increase in tumor volume, whether with or without laser (Fig. 8c). The tumors in the PG-injected mice (-NIR) grew similarly to the ones treated with PBS-treated mice, while the tumor volume of the NIR-treated PG groups had a slower growth due to the low-temperature rise of PG hydrogel. Similarly, the tumor volume of the PGC and PGCA-injected groups without NIR irradiation showed rapid growth. In contrast, PGC and PGCA-treated groups with laser irradiation showed a very effective shrinkage and destruction of the tumor tissue due to the localized CuO-mediate hyperthermia, which induced cancer cell apoptosis or necrosis. The relative tumor volume meaningfully decreased for the PGC and PGCA treated groups exposed to the NIR. The photographs of the tumor tissues of different groups are shown in Fig. 8d. H&E-stained tumor tissues of the PGC (+NIR) and PGCA (+NIR)-treated groups exhibited significant necrosis and nuclear damage of tumor cells compared to other groups (Fig. 8e). The expression of the Ki-67 antigen in tumor cells was significantly reduced in the PGC (+NIR) and PGCA (+NIR)-treated groups, indicating suppressed proliferation of tumor cells under photothermal treatment. However, the other groups including, PBS (±NIR), PG (±NIR), PGC (-NIR), and PGCA (-NIR) showed high Ki-67 expression. Furthermore, except for the PGC (+NIR) and PGCA (+NIR) groups, the VEGF expression was considerably high in all tumor-bearing groups (Fig. S17). This is attributed to both transformed melanocytes and tumor-induced hypoxia, which lead to promoted angiogenesis in order to facilitate the supply of nutrients and oxygen to the tumor cells, ultimately promoting cancer progression [54,55]. However, tumor-bearing mice treated with CuO-containing groups under laser irradiation exhibited lower VEGF expression, confirming the inhibited angiogenesis and tumor ablation ability of PGC and PGCA hydrogels under NIR irradiation. Throughout the 15-day observation period, all mice did not exhibit significant body-weight loss, suggesting negligible side effects (Fig. S18a). All the data supported the notion that CuO nanosheet-induced hyperthermia under laser irradiation could significantly suppress tumor growth, highlighting PGC and PGCA hydrogels as potential therapeutic agents for inducing tumor cell death.

### 2.9 In vivo wound healing effect

**Fig. 9:**
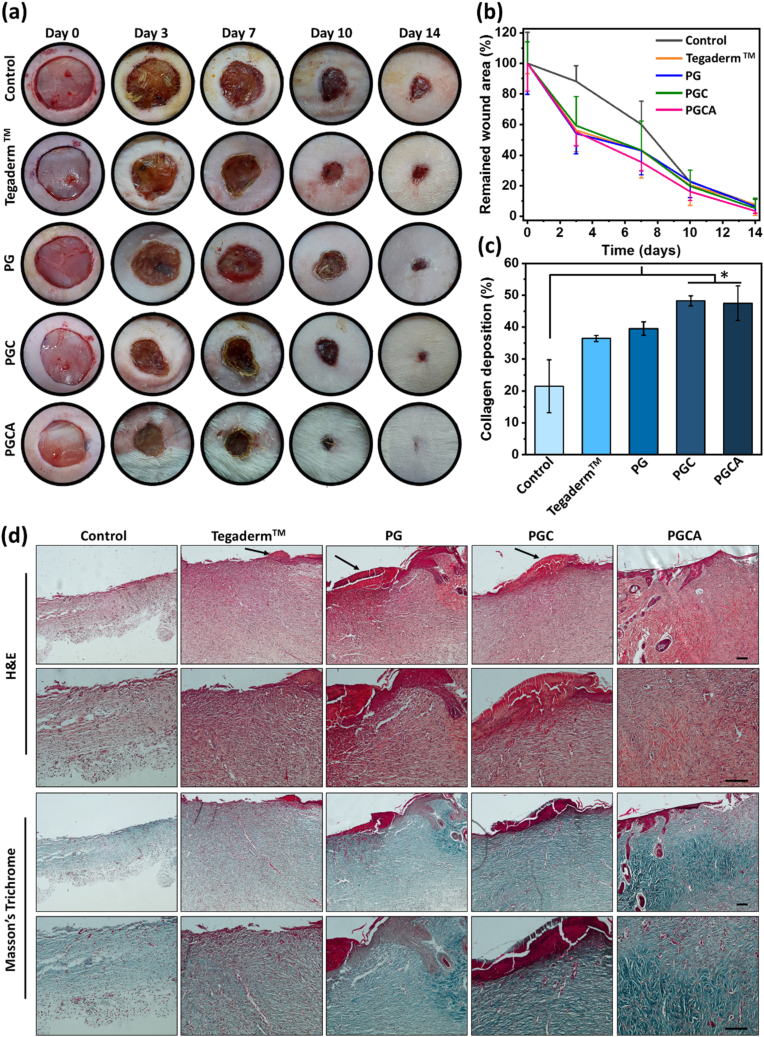
(a) Macroscopic photographs of the wound area of the control, Tegaderm™, PG, PGC, and PGCA-treated groups on days 0, 3, 7, 10, and 14. (b) Remained wound area of different formulations on days 0, 3, 7, 10, and 14 (N = 4). (c) Collagen deposition of different groups at day 14. Data are presented as mean ± SD (N = 3; *p < 0.05). (d) Representative images of H&E and Masson's trichrome-stained tissues on day 14 post-surgery. Scabs are shown with black arrows. Scale bar = 200 μm.

To assess the wound healing efficacy of PG, PGC, and PGCA hydrogels, the full-thickness cutaneous wound model was developed on the back of rats (Fig. S19). Digital photographs were taken to monitor the wound healing process for 14 days. As shown in Fig. 9a, on day 3, the wound area of the PGCA-treated group was considerably reduced in comparison to the control and Tegaderm™-treated groups. The relative remained wound area on day 7 was 35.6 % for the PGCA-treated group, while it was 60 %, 42.8 %, 43.1 %, and 43.4 % for the control, Tegaderm™, PG, and PGC-treated groups, respectively. Although the PGCA-treated wounds almost closed after 14 days of screening, the wounds of the control and Tegaderm™ groups remained open with scars and cell debridements. Therefore, the quantitative studies demonstrated the best wound closure performance for the PGCA hydrogel, which would be a promising candidate for wound defects and tissue regeneration (Fig. 9b).

The wound healing efficacy of hydrogels was further studied by the histological assessments on day 14 of screening (Fig. 9d). The PGCA-treated group remarkably demonstrated thicker granulation tissue and the most integrated epidermis structure without any scabs in comparison to the other groups, which confirmed the full healing of the dermis tissue. Moreover, the PGCA-treated group represented a reduced neutrophil infiltration, while a large number of neutrophils were observed for the control group. Also, more blood vessels were observed in the PGCA-treated group which could deliver more nutrients and oxygen to the damaged site, resulting in much fibroblast proliferation and denser organized collagen fiber deposition. The generation of blood vessels is presumably attributed to the stimulatory effect of hyperthermia and CuO nanosheets on angiogenesis through the induction of angiogenesis-related genes including VEFG and stabilizing hypoxia-inducible factor (HIF-1a) [[56], [57], [58]]. The quantitative analysis of Masson's trichrome-stained tissues confirmed the intense collagen deposition and thicker epidermis layer formation in the PGCA-treated group compared to the other groups (Fig. 9c and Fig. S20). The antibacterial potential of CuO nanosheets and the regenerative and smoothing effect of released allantoin (Fig. S21) along with the extracellular matrix-mimicking effect of gelatin, as well as the good swelling behavior of the hydrogel resulted in accelerated wound recovery.

### 2.10 Adhesive properties and in vivo wound closure effect

**Fig. 10:**
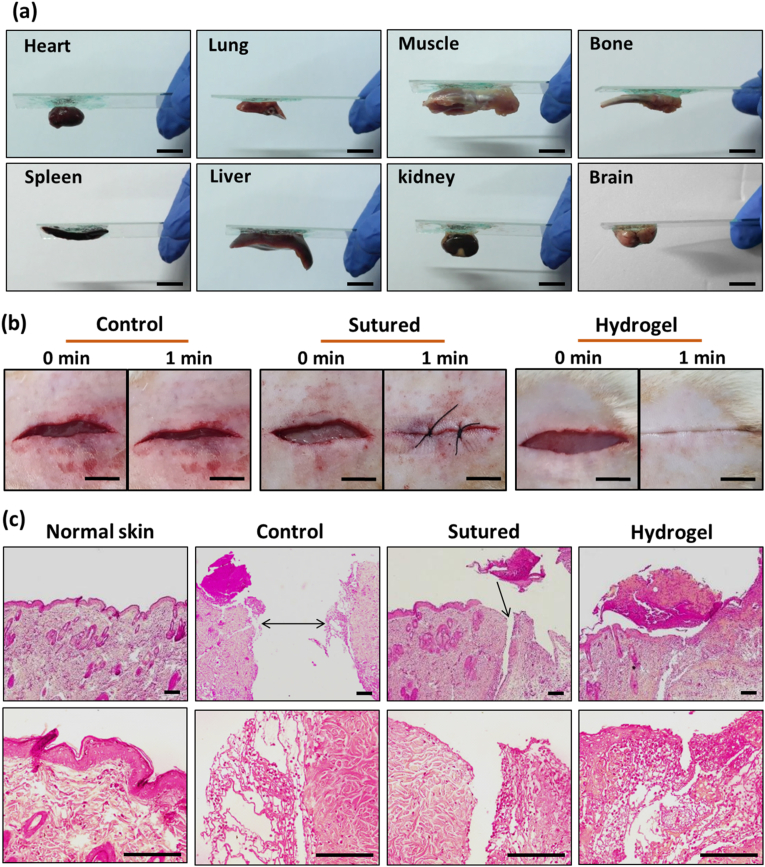
(a) Macroscopic images of adhered PGCA hydrogel to various rat tissues, including heart, lung, muscle, bone, spleen, liver, kidney, and brain. Scale bars = 20 mm. (b) In vivo wound closure assessment in the control, sutured, and PGCA hydrogel groups in a full-thickness cutaneous incision at 0 and 1min post-treatment. Scale bars = 5 mm. (c) H&E-stained skin tissues on day 3 of the study. Scale bars = 200 μm.

The adhesive properties of PGCA hydrogel were evaluated using biological tissues, including heart, lung, muscle, bone, spleen, liver, kidney, and brain as well as in different substrates, including glass, polytetrafluoroethylene (PTFE), polypropylene (PP), stone, polydimethylsiloxane (PDMS), rubber, stainless steel, aluminum, and wood. The bioadhesive property of the hydrogel resulted in its high interfacial adhesion capacity to both biological tissues and substrates (Fig. 10a and Fig. S22). Moreover, good bioadhesiveness of the hydrogel to the wound site was also shown during wound twisting (Fig. S23). To further demonstrate the role of bioadhesivity in wound healing, the closure effect of PGCA hydrogel was determined in the control, sutured, and hydrogel-treated group using a full-thickness dorsal skin injury. The macroscopic images showed a strong closure after bringing bilateral tissues together within 1 min in hydrogel-treated rats, while the wounds of non-treated groups remained open (Fig. 10b, Video S2). Also, the hydrogel-treated group showed rapid and easy alignment of wound edges by a simple procedure without redness in comparison to surgical sutures. The histological assessment on the 3rd day of treatment showed significant diversities in the structure of skin among different treated groups (Fig. 10c). The tissue integrity and aligned repositioning with the formation of granulation tissue were observed in the hydrogel-treated group due to cell migration. However, in the sutured group, a slight extent of granular tissue was observed along with a remained gap. The PGCA-treated group also represented more skin appendages in comparison to sutured group. Hence, adhesive PGCA hydrogel is an excellent contraction agent for wound sites and an alternative candidate to traditional sutures, which can also accelerate the wound healing process.

### 2.11 In vivo anti-post-operative adhesion evaluation

Post-operative wounds, as injuries that occur as a result of a surgical procedure or photothermal tumor ablation, require proper care to promote healing and prevent complications of postsurgical adhesion due to abnormal connections of wound area to surrounding tissues and organs. As we know, wounds create in the internal and abdominal organs after tumor ablation, where the risk of post-treatment adhesion is very high. Therefore, surgical resection of the tumor should be combined with a multifunctional formulation that presents wound repair effect and a desirable preventing effect on post-operation adhesion. Therefore, to present the potential of our formulation for combined photothermal tumor ablation and adhesion prevention, we performed in vivo anti-adhesion assays.

**Fig. 11:**
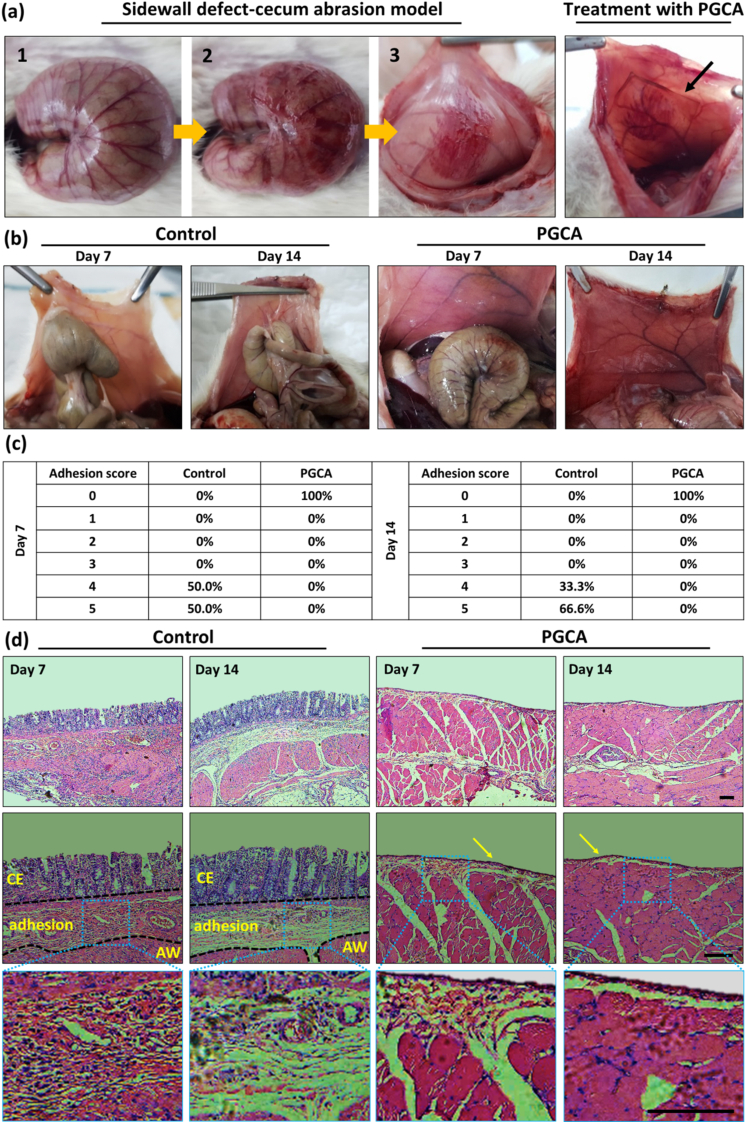
(a) The photographs of constructing a sidewall defect-cecum abrasion model and placing the PGCA hydrogel film onto the damaged site. The black arrow shows PGCA film on the wound area. (b) Digital photographs of adhesion assessment of the control and PGCA groups on days 7 and 14 post-surgery. Hydrogels were disappeared from the implantation site, demonstrating their biodegradation. (c) The adhesion scores of control and PGCA film on days 7 and 14 according to the standard adhesion scoring system are explained in the Table S1 of the Supplementary Information (N = 6). (d) H&E stained abdominal wall defect of control and PGCA groups on days 7 and 14 with different magnifications. CE: cecal mucosa; AW: abdominal wall. Arrows show the mesothelial layer. Scale bar = 200 μm.

Although there are many physical barriers for preventing abdominal adhesions, little attention has been paid to formulating a system that accelerates wound healing after surgery in the abdominal area while preventing post-operative adhesion [59]. Therefore, taking advantages of film-forming property of PGCA formulation (Fig. S12), the anti-adhesive effect of the PGCA film was tested using a sidewall defect-cecum abrasion model as shown in Fig. 11a. The photographs of abdominal adhesion of control and PGCA groups are shown on days 7 and 14 after treatment (Fig. 11b). No adhesion was observed in the PGCA film group on days 7 and 14 of the study, indicating the high performance of PGCA film in preventing postsurgical abdominal adhesion. Also, the healing of the injured abdominal defects with no sign of scar and recovery of the damaged cecum was identified. In contrast, on the 7th day of the study, all rats of the control group demonstrated severe abdominal adhesion and 50 % of rats scored 4 and the other half scored 5 for abdominal adhesion according to the standard adhesion scoring system (Fig. 11c). Also, after 14 days 66.6 % of the control group demonstrated a score of 5, and 33.3 % scored 4, with more vascularized and compact adhesion even in the undamaged parts of the cecum.

According to histological images, the PGCA-treated group was able to prevent adhesion and re-mesothelialized the injured abdominal wall (Fig. 11d). However, some fibroblasts and inflammatory cells were still observed in the subjacent layers on day 7, which were reduced on day 14. As for the control group, the presence of fibroblasts, inflammatory cells, and collagen deposition in the tissue was visible on day 7 where the cecum and abdominal wall were connected. Moreover, on day 14 the thicker adhesion region with more collagen deposition was observed for the control group. Therefore, the results confirmed the strong effectiveness of PGCA film in preventing peritoneal adhesions in a sidewall defect-cecum abrasion model with a desirable wound healing effect.

## 3 Conclusion

In summary, a multifunctional therapeutic hydrogel was successfully developed by crosslinking reaction between PMVE/MA and gelatin and loading of CuO nanosheets and allantoin. This hydrogel exhibited time-dependent viscosity enhancement, allowing it to be injectable during the initial hours and efficiently perform PTT later to ablate cancer cells through the NIR-responsive activity of CuO nanosheets. Additionally, the synthesized CuO nanosheets demonstrated inherent antibacterial effects against both gram-positive and gram-negative bacteria, as well as MRSA infection, providing a synergistic effect in preventing microbial infections during the treatment of melanoma and postsurgical wounds. Moreover, the PGCA hydrogel applied to the wound bed could accelerate the healing process owing to the angiogenesis ability of CuO nanosheets in contribution to the re-epithelialization and collagen deposition activity of allantoin. Taking the advantage of the film-forming ability of the hydrogel, it could also be utilized as a physical barrier to prevent abdominal adhesion post-operation while keeping the wound healing properties. Furthermore, due to the adhesive property, the hydrogel could be utilized for sutureless wound closure with less scar formation. Overall, this biocompatible multifunctional hydrogel offers a promising approach for simultaneous treatment of skin-tumor therapy, infection removal, and accelerated wound healing with minimal invasiveness and high performance.

## Experimental section

The experimental details are reported in the **Supplementary Information**.

## CRediT authorship contribution statement

**Vahideh Nosrati-Siahmazgi:** Writing – original draft, Validation, Software, Resources, Methodology, Investigation, Formal analysis, Conceptualization. **Samin Abbaszadeh:** Software, Methodology, Formal analysis, Data curation. **Kiyan Musaie:** Software, Methodology, Investigation. **Mohammad Reza Eskandari:** Validation, Resources. **Saman Rezaei:** Methodology, Data curation. **Bo Xiao:** Validation, Resources. **Fatemeh Ghorbani-Bidkorpeh:** Validation, Resources. **Mohammad-Ali Shahbazi:** Writing – review & editing, Supervision, Resources, Project administration, Conceptualization.

## Declaration of competing interest

The authors declare that they have no known competing financial interests or personal relationships that could have appeared to influence the work reported in this paper.

## Data Availability

Data will be made available on request.
