# Supplementary material for: NIR‐Responsive injectable hydrogel cross-linked by homobifunctional PEG for photo-hyperthermia of melanoma, antibacterial wound healing, and preventing post-operative adhesion
Source: Mater Today Bio. 2024 Apr 24;26:101062. doi: 10.1016/j.mtbio.2024.101062 (PMC11066557; doi:10.1016/j.mtbio.2024.101062)
Supplement: Multimedia component 1 [file mmc1.docx]

NIR‐Responsive Injectable Hydrogel Cross-Linked by Homobifunctional PEG for Photo-Hyperthermia of Melanoma, Antibacterial Wound Healing, and Preventing Post-Operative Adhesion

Vahideh Nosrati-Siahmazgi^1^, Samin Abbaszadeh^2^, Kiyan Musaie^3^, Mohammad Reza Eskandari^4^, Saman Rezaei^3^, Bo Xiao^5^, Fatemeh Ghorbani-Bidkorpeh^6^, Mohammad-Ali Shahbazi^1,3,7,^*

^1^ Department of Pharmaceutical Biomaterials, School of Pharmacy, Zanjan University of Medical Science, 45139-56184 Zanjan, Iran

^2^ Department of Pharmacology, School of Medicine, Zanjan University of Medical Sciences, 45139-56111 Zanjan, Iran

^3^ Department of Biomaterials and Biomedical Technology, University Medical Center Groningen, University of Groningen, Antonius Deusinglaan 1, 9713 AV Groningen, Netherlands

E-mail: [m.a.shahbazi@umcg.nl](mailto:m.a.shahbazi@umcg.nl)

^4^ Department of Pharmacology and Toxicology, School of Pharmacy, Zanjan University of Medical Science, 45139-56184 Zanjan, Iran

^5^ State Key Laboratory of Silkworm Genome Biology, College of Sericulture, Textile and Biomass Sciences, Southwest University, Chongqing, 400715 China

^6^ Department of Pharmaceutics and Pharmaceutical Nanotechnology, School of Pharmacy, Shahid Beheshti University of Medical Sciences, Tehran, Iran

^7^ W.J. Kolff Institute for Biomedical Engineering and Materials Science, University of Groningen, Antonius Deusinglaan 1, 9713 AV Groningen, The Netherlands

**Keywords:** skin cancer, photothermal therapy, copper oxide nanoparticle, wound repair, abdominal adhesion

**1. Experimental Section**

**1.1. Synthesis of CuO Nanosheets**

In a typical synthesis process, a polyvinylpyrrolidone (PVP) (Sigma-Aldrich, USA) solution with a concentration of 0.5 wt% was prepared in a volumetric flask. Then, 1.45 g of copper (П( nitrate trihydrate [Cu(NO_3_)_2_.3H_2_O] (>95%, Merck, Germany) was added to 60 ml of PVP solution under vigorous stirring. Next, 15 ml of NaOH (1 M) was added to the previous mixture to make the solution alkaline. The mixture was stirred at 60 °C for 4 h, and then centrifuged at 8500 rpm for 4 min. The sediment was washed three times with distilled water to remove the by-products and impurities, centrifuged at 8500 rpm for 4 min, and finally dried overnight in the oven at 60 ˚C for later usage.

**1.2. Fabrication of Hydrogels**

To prepare the primary polymer solution, 10% w/v of poly(methyl vinyl ether-alt-maleic anhydride) (PMVE-MAH; Sigma-Aldrich, USA; average molecular weight 1,080,000 Da) was prepared in distilled water under vigorous stirring in a volumetric flask for 10 h at 80 °C to hydrolyze it and obtain a clear solution of poly(methyl vinyl ether-alt-maleic acid) (PMVE-MA). To fabricate the PG hydrogel, 400 μl of poly (ethylene glycol) diglycidyl ether (PEGDGE, Sigma-Aldrich, Japan), as the cross-linker, was added to 1.5 ml of the obtained PMVE-MA solution and mixed well. Subsequently, 500 μl of gelatin solution (10% w/v, porcine skin, SLBZ4132, Sigma-Aldrich, USA) was prepared under constant stirring at 50 °C in a water bath and added to the above-mentioned mixture to obtain the hydrogel network after leaving at room temperature for 3 h.

Moreover, to prepare PGC hydrogel, the synthesized CuO nanosheets were added to the PMVE-MA solution under vigorous stirring to obtain the final concentration of 400 μg ml^-1^ of the CuO nanosheets in the hydrogel following the above-mentioned steps. PGCA hydrogel was also prepared by the addition of allantoin (≥98%, Sigma Aldrich, USA) in the PMVE-MA solution containing CuO nanosheets to obtain the final concentration of 400 μg ml^-1^ in the hydrogel following the above-mentioned procedure.

**1.3. Characterization of CuO Nanosheets**

The morphological characterization of the synthesized CuO nanosheets was examined with transmission electron microscope (TEM, Philips EM 2085, USA) and field emission scanning electron microscope (FE-SEM, TESCAN MIRA3, Czech Republic) equipped with energy-dispersive X-ray (EDAX) spectroscopy to further evaluate the elemental composition of CuO nanosheets. The zeta potential of CuO nanosheets was measured using a zeta-sizer (Horiba Jobin Jyovin, SZ-100z, Japan) at room temperature. The ultraviolet-visible (UV-Vis) absorption spectra was also assessed by a spectrometer (Genesys 10-S, USA) in the scan range of 400-900 nm.

**1.4.** Characterization of Hydrogels

*1.4.1.* *Gelation Time, porosity and morphology of the hydrogel*

The gelation time of PGCA hydrogel was investigated by the tube inversion method ^1-2^. First, 1 ml of PGCA hydrogel with and without PEGDGE was immersed in a water bath at 25 °C and 70 °C at different time points and then inverted and the time that the hydrogel stopped flowing was considered as gelation time.

For the calculation of porosity, we conducted solvent displacement method. The hydrogels (with and without CuO) were first prepared and freeze-dried. Next, the freeze-dried samples, with known weight, were immersed in absolute ethanol and kept until complete saturation in ethanol. After the samples were drowned, the percentage of the porosity was calculated using the Equation (1):

$\mathrm{Porosity}\left( \% \right)=\frac{(Ws-Wd)}{\mathrm{Vs}\rho ethanol}$ $(1)$

where, Ws is the weight of the drowned sample after immersing in absolute ethanol, Wd is the weight of the freeze-dried sample, Vs is the volume of the sample after freeze-drying and ρ is the density of ethanol. The morphology of the freeze-dried hydrogels and their pores were also visualized by SEM by excising thin sections of the samples and coating with a thin layer of gold.

*1.4.2* *Initial Water Content, Swelling Ratio, and Water Retention*

To assess the initial water content, the initial wet weight and the weight of the completely dried PG, PGC, and PGCA hydrogels were measured. The initial water contents (IWC) were calculated using the Equation (2):

$$\mathrm{IWC} (\%)=\frac{Initial wet weight of the hydrogels-Weight of dried hydrogels}{Initial wet weight of the hydrogels}\times100 (2)$$

To evaluate the swelling ratio, dried PG, PGC, and PGCA hydrogels were weighted (W_d_) and then immersed in 50 ml of phosphate buffer saline (PBS; pH 7.4) at 37 ^°^C to mimic the physiological conditions. After removing the excess water with a filter paper, the weight of the swelled hydrogels (W_s_) was measured at pre-determined time points (0.25, 0.5, 1, 2, 4, 8, and 24 h), until the weight of hydrogels reached to the steady state. The swelling ratio of hydrogels was calculated using Equation (3):

$$Swelling ratio (\%)=\frac{W_{s}-W_{d}}{W_{d}}\times100 (3)$$

To determine the water retention capacity, the dried PG, PGC, and PGCA hydrogels were incubated in PBS (pH 7.4) at 37 ˚C for 48 h, and then the weight of hydrogels were monitored at different times (0.5, 1, 2, 4, 6, 8, 12, 24, 48, and 72 h) in the open air. The water retention ratios were calculated according to Equation (4):

$$Water retention ratio (\%)=\frac{W_{t}-W_{0}}{W_{0}}\times100 (4)$$

where W_t_ and W_0_ represent the weight of hydrogel at time t and the weight of the dry sample, respectively ^3^. All experiments were carried out at least in triplicate.

*1.4.3. Yield and degradation*

To evaluate the yield, PG, PGC, and PGCA hydrogels were completely dried in the oven at 60 ^°^C for 72 h and weighed. The yield ratios of the hydrogels were calculated using Equations (5), (6), and (7):

$$Yield\% \left( \mathrm{PG} \right)=\frac{Weight of dried PG hydrogel}{The initial weight of ((PMVE-MA)+PEGDGE+Gelatin)}\times100 (5)$$

$$Yield\% \left( \mathrm{PGC} \right)=\frac{Weight of dried PGC hydrogel}{The initial weight of ((PMVE-MA)+ PEGDGE+ Gelatin+CuO)}\times100 (6)$$

$$Yield\% \left( \mathrm{PGCA} \right)=\frac{Weight of dried PGCA hydrogel}{The initial weight of ((PMVE-MA)+PEGDGE+Gelatin+CuO+allantoin)}\times100 (7)$$

To determine the percentage of degradation, PG, PGC, and PGCA hydrogels were completely and weighted (W_initial_) and immersed in 50 ml of PBS solution (pH 7.4) at 37 °C. After 8 and 24 h, the hydrogels were taken out from the PBS solution and dried in the oven at 70 ºC for 48 h (W_d_). The percentage of degradation was calculated using the Equation (8):

$$Degradation\%=\frac{(W_{\mathrm{Initial}}-W_{d})}{W_{\mathrm{Initial}}} \times100 (8)$$

*1.4.4. ATR-FTIR, XRD, TGA, and DTG Analysis*

Attenuated total reflectance Fourier transform infrared (ATR-FTIR, Thermo Nicolet Avatar, USA) was conducted at room temperature in the spectral range of 4000 to 600 cm^-1^ and the resolution of 4.0 cm^-1^ to investigate the chemical composition of materials and successful preparation of hydrogels. Moreover, to determine the crystalline structure of the specimens, an X-ray diffractometer (XRD, Philips PW1730, Netherlands) was used in the 2θ range of 10-80°. Thermal stability and weigh loss (%) of the initial components and hydrogels were also evaluated by thermogravimetric analysis (TGA; SDT-Q600, USA) and derivative thermogravimetry (DTG) analysis under argon atmosphere with the temperature range of 30 to 800 °C and heating rate of 10 °C min^−1^.

*1.4.5. Rheological Measurements and Injectability*

To determine the viscosity of PG and PGC hydrogels, the viscosity as a function of shear rate was assessed by varying the shear rate from 10 s^-1^ to 160 s^-1^ using a rheometer (R/S plus, Brookfield, Canada) with parallel plate geometry (25 mm diameter) after 5 min and 24 h of preparing the hydrogels and keeping at 25 °C. Moreover, the rheological properties of PG and PGC hydrogels were explored using an MCR 302 rheometer (Anton paar, Austria) via three different test methods, including 1) Frequency sweep measurements at the frequency range of 0.1 to 100 rad s^-1^ with a constant strain of 0.1% to determine the storage (G′) and loss (G″) moduli; 2) Strain sweep test from 0.01% to 1000% with a constant frequency of 10 rad s^-1^ to evaluate the linear viscoelastic region; and 3) The alternate-step strains test to detect the self-recovery property of hydrogels by switching from small strain (0.1%) to large strain (200%) in 5 cycles with 100 s intervals at the frequency of 10 rad s^−1^. All the three above tests were conducted after 48 h and 10 days of hydrogel fabrication and storing at room temperature.

The force needed for the injection of hydrogels was evaluated using a mechanical testing machine (SANTAM, STM 5, Iran) and a 500 N load cell with a speed rate of 1 mm s^-1^. PGCA hydrogel was loaded into 10-ml syringes with 21-gauge needles 2 and 4 h after hydrogel fabrication at room temperature. Displacement and force were recorded until the hydrogels were extruded completely and the force was plotted against displacement.^4^ In addition, to visualize the injectability, PGC hydrogel was added into a 5-ml syringe with a 21-gauge needle 2 h after preparation, and was manually injected into a glass filled with deionized water (DW). Further, the hydrogel was injected to write “PN” and photographed.

*1.4.6. Mechanical Studies*

The mechanical properties of cylindrical shaped PG, PGC, and PGCA hydrogels (18 mm high×17 mm in diameter) were studied 10 days after preparation by SANTAM (STM-5, Iran) compression test machine (500 N load cell) with a speed of 10 mm min^-1^. The compression resilience of the hydrogels was recorded at 60% strain and unloaded to 0% strain, up to 10 cycles. Then, it was loaded to 75% strain again to assess the compression strength of the hydrogels. Each process was carried out 3 times at room temperature.

*1.4.7. Moldability and stretchability*

To examine the moldable and stretchable properties, the fabricated hydrogel was poured into the rectangle mold with the height of 3 mm and was placed in the ambient temperature for 2 days to dry completely. Then the macroscopic images were taken after film rolling, adhering to the skin, bending and removing, and also stretching the film.

**1.5. In Vitro Photothermal Performance of CuO Nanosheets and Hydrogels**

The photothermal activity of CuO nanosheets, PG, and PGC hydrogels was evaluated using 808 nm near infrared (NIR) laser. The suspensions of CuO nanosheets (100, 200, and 400 µg ml^-1^) were exposed to 808 nm NIR laser with different power densities (0.5, 1, and 1.5 W cm^-2^) for 10 min. Also, PGC hydrogel with concentrations of 100, 200, and 400 µg ml^-1^ of CuO nanosheets were studied under same condition. The temperature rise of the samples was monitored using a digital thermal imaging camera (ht-02, hti, Italy, with an accuracy of 0.15 °C) at 1 min intervals. To evaluate the photothermal stability, PGC hydrogel with 400 µg ml^-1^ of CuO nanosheets was exposed to 808 nm NIR irradiation for 10 min at 1.5 W cm^-2^ and then cooled naturally in 14 min to the room temperature. The heating/cooling process repeated for four cycles and the temperature was monitored.

*1.5.1. Photothermal Conversion Efficiency*

To evaluate the photothermal conversion efficiency (η), 1 ml of PGCA hydrogel was irradiated by an 808 nm laser with a power density of 1.5 W cm^-2^ for 10 min, and then the laser turned off until the sample cooled to reach the ambient temperature. The temperature was monitored every 30 s in the cooling phase for 14 min. The η of CuO nanosheets incorporated in PGCA hydrogel was determined according to Equation (9) ^5^:

$ƞ=\frac{hA(T_{\mathrm{Max}}-T_{\mathrm{Surr}})-Q_{\mathrm{Dis}}}{I (1-{10}^{-A808})} (9)$

Where, h represents the heat transfer coefficient, and A referrs to the surface area of the quartz cell. T_Max_ (55.3 °C) and T_surr_ (28°C) were the maximum temperature of the hydrogel under irradiation and ambient temperature, respectively. Therefore, the temperature changes (T_Max_-T_Surr_) was calculated to be 27.3 °C. Q_Dis_ represented the heat dissipation of the light absorption by DW and quartz cell. The laser power density (I) was 1500 mW cm^-2^, and the absorbance of CuO nanosheets at 808 nm (A_808_) was 1.5. Afterward, hA value was acquired using Equation (10):

$$\tau_{s}=\frac{m_{D}\times C_{D}}{\mathrm{hA}} (10)$$

The time constant (τ_s_) value was acquired from the slope of the linear time data from the cooling state versus negative natural logarithm of the driving force temperature (Figure 4i) using Equation (11):

$t=- \tau$_s_ ln(θ) (11)

And θ was calculated from Equation (12):

$$\theta=\frac{T-T_{\mathrm{Surr}}}{T_{\mathrm{Max}}- T_{\mathrm{Surr}}} (12)$$

Where, m_D_ and c_D_ were the mass (0.7 g) and heat capacity (4.2 J g^-1^ °C^-1^) of the DW present in the PGCA hydrogel, respectively. Accordingly, the hA value was calculated to be 11.4 mW °C^-1^. Then, Q_Dis_ was calculated using Equation (13):

$Q_{\mathrm{Dis}}=\frac{m_{D}\times c_{D}(T_{\mathrm{Max}}-T_{\mathrm{Surr}})}{\tau_{s}} (13)$

m_D_ stands for the mass of DW (1 g) under 10 min 808 nm NIR irradiation with power density of 1.5 W cm^-2^. T_max_, T_Surr_ and τ_s_ for DW were 29.9 °C, 23.3 °C, and 519.5, respectively. The value of Q_Dis_ was 54 mW, and thereupon η value of the CuO nanosheets was calculated 23% according to the Equation (9).

**1.6. Hemocompatibility of the Hydrogels**

The hemocompatibility of PG, PGC, and PGCA hydrogels was evaluated by hemolysis assay in vitro ^6^. To obtain erythrocytes, PBS buffer (pH 7.4) was added to anti-coagulated fresh human whole blood with 1:2 ratio of the RBC to the PBS buffer and then was centrifuged at 3000 rpm for 6 min. To collect purified erythrocytes, the precipitated solution was washed 5 times with PBS as the ratio mentioned above and was further diluted to a ﬁnal concentration of 5% (v/v). Next, the dried hydrogel (0.5, 1, 1.5, and 2 mg ml^-1^) was dispersed in 800 μl of PBS buffer (pH 7.4) and then mixed with 200 μl of RBC suspension. After incubation at room temperature for 2, 4, 8 and 24 h, the samples were centrifuged at 4000 rpm for 5 min. For each sample, 150 µl^-1^ of the supernatant were transferred into a 96-well plate and the absorbance of the solutions was recorded at 540 nm using a microplate reader (Inﬁnite M200, Austria). DW and PBS buffer were employed as the positive and negative control, respectively. The non-hemolyzed percentage of the hydrogels was calculated using the equation (14):

$$Non-hemolyzed RBC \left( \% \right)=\left( 1-\frac{Absorbance of \left( sample- negative control \right)}{Absorbance of (positive control-negative control} \right)\times100 (14)$$

**1.7. Cell viability studies**

For the cytotoxicity study of different hydrogels, NIH/3T3 and HDFa normal fibroblasts as well as B16F10 cancer cells were seeded at a density of 8×10^4^ cells per well in a 24-well Transwell® plate. Following overnight attachment, freshly prepared PG, PGC, and PGCA hydrogels (350 µl final volume) were added to the upper chamber of the Transwell®. The samples were then incubated at 37 ˚C for 24 and 48 h. A negative control group was established using a cell culture medium without any tested materials. After treatment, cell viability was assessed using the CellTiter-Glo® luminescent assay, which involved transferring the reagents and cells to a 96-well plate. The luminescence intensity of the samples was measured using a multimode microplate reader (Varioskan™, ThermoFisher, USA). All samples were tested in four replicates to ensure accuracy and reproducibility of the results.

Moreover, the effects of in vitro PTT were evaluated in B16F10 cells. The cells were seeded in a 96-well plate at a density of 1.5×10^4^ cells per well one day prior to the experiment. Subsequently, the cell culture medium in each well was replaced with 75 µl of the Hank’s balanced salt solution (HBSS)−(4-(2-hydroxyethyl)-1 piperazineethanesulfonic acid (HEPES) buffer (pH 7.4), and 75 µl of PG, PGC, and PGCA hydrogels were injected into the wells. Cells treated with PBS injection served as controls. The cells were then exposed to a near-infrared (NIR) laser at a wavelength of 808 nm, with a power density of 1 W cm^-2^ for 10 min. After the laser exposure, the hydrogel and HBSS-HEPES buffer were carefully removed from the wells, and cell viability was assessed using the CellTiter-Glo® luminescent assay following the manufacturer's protocol. The luminescence intensity, which is indicative of cell viability, was measured using a Varioskan™ LUX multimode microplate reader to obtain quantitative results. The samples were tested in 4 replicates.

**1.8. In Vivo Toxicity of Hydrogels**

The acute toxicity of PG, PGC, and PGCA hydrogels was determined in rats. For this purpose, 16 male Sprague-Dawley rats with a weight range of 200–220 g were randomly divided into four groups (N=4). At first, the animals were anesthetized by intraperitoneal (IP) injection of 0.25 ml of ketamine (50 mg ml^−1^)-xylazine (20 mg ml^−1^) cocktail (6:4 v/v). After shaving the back of rats, 500 µl of the hydrogels was subcutaneously injected into the back of rats by a 22-gauge needle except for the control group, which received no treatment. On day 14, 2 ml of blood was collected from animals in all groups for blood biochemistry and hematological assessment. Next, the rats were euthanized and the main organs including liver, kidney, spleen, and the skin at the site of injection were collected and fixed in 4% formaldehyde for 8 h, followed by embedding in paraffin and sectioning by microtome to slides before hematoxylin and eosin (H&E) staining. The slides were visualized by an optical microscope (Olympus BX61, Japan).

**1.9.** Antibacterial Studies

*1.9.1. In Vitro Antibacterial Test*

To investigate the antibacterial activity of CuO nanosheets, and the hydrogels, *Staphylococcus aureus* (*S. aureus*; ATCC 25923) and *Escherichia coli* (*E. coli*; ATCC 25922) were selected as the representative of Gram-positive and Gram-negative bacteria, respectively. Both bacteria were cultivated in sterilized nutrient at 37 °C under continuous shaking at 150 rpm overnight. Next, 20 µl of bacterial suspension with a concentration of 10^6^ CFU ml^−1^ (diluted with normal saline 0.9% w/v) was added into 1 ml of an aqueous dispersion of CuO nanosheets (400 μg ml^-1^), or the hydrogels. After 2 h of incubation at 25 °C, samples were diluted 30 times and 100 μl of the diluted media were uniformly spread onto agar plates using sterile swabs and incubated for 18 h at 37 °C. Normal saline was considered as a control group according to the same protocol and the bacterial growth was assessed on the agar plates. All samples were studied in triplicate.

*1.9.2. In Vivo Antibacterial Test*

The antibacterial activity of the PG and PGC hydrogels was also investigated in vivo by a murine subcutaneous abscess model. Firstly, the backs of the mice were shaved and disinfected, and then, 200 µl of Methicillin-resistant *Staphylococcus aureus* (MRSA; 10^7^ CFU ml^−1^) in normal saline was injected subcutaneously into both left and right sides of the dorsal surface of the animals. An obvious subcutaneous infected abscess was observed 24 h after injection. The mice (N=4) were randomly divided into ten groups: non-infected, infected (without any treatment), PBS (-NIR), PBS (+NIR), CuO (-NIR), CuO (+NIR), PG (-NIR), PG (+NIR), PGC (-NIR), and PGC (+NIR), and 100 µl of each sample was directly injected into the infected site. The 808 nm laser (1.5 W cm^−2^) was applied to the subcutaneous abscess of the NIR positive groups 2 h after the injection for 6 min. Using an IR thermal camera, the temperature was controlled so that it did not exceed 52 ºC. After 13 days, all mice were sacrificed and their skins were photographed and excised for further histological and immunohistochemistry analysis. The skin of the infected mice after abscess formation (24 h after the injection of the bacteria) group was also excised and stained as the positive control. Subsequently, the abscesses were completely removed and immersed in 8 ml of physiological saline solution and were diluted 200 times and then 100 μl of the diluted dispersion was uniformly spread onto agar plates using sterile swabs and incubated for 18 h at 37 °C to monitor bacterial growth.

**1.10. In Vivo Photothermal Tumor Therapy**

In order to evaluate the in vivo photothermal effect of PG, PGC, and PGCA hydrogels for cancer therapy, a subcutaneous melanoma tumor model was established. For this aim, 2×10^6^ of B16-F10 cells in 100 µl PBS were injected subcutaneously in the hind part of each BALB/c mice (Female, 5 weeks old). When the tumor volume of mice reached about 300 mm^3^, the mice were randomly assigned into eight groups (N=8) and received 100 µl of different treatments subcutaneously at the tumor site as follows: PBS (-NIR), PBS (+NIR), PG (-NIR), PG (+NIR), PGC (-NIR), PGC (+NIR), PGCA (-NIR), and PGCA (+NIR). Then, the entire tumor area of each mice in NIR groups were exposed to the 808 nm laser irradiation (1.5 W cm^-2^, 6 min). The temperature was adjusted to 52 ˚C and was monitored by a thermal imaging camera. The tumor volumes were measured on days 0, 3, 6, 9, 12, and 15 by a digital caliper and calculated according to the following equation: V (mm^3^)=(tumor length)×(tumor width)^2^/2. Moreover, the body weights were monitored during the experiment. After 15 days, the macroscopic images were taken from the tumor sites of each mice. Then, the mice were sacrificed and the tumors were excised and photographed. The tumor tissues were routinely fixed in 4% formaldehyde, embedded in paraffin, and sectioned. Histological analysis was explored by H&E, Ki67, and vascular endothelial growth factor (VEGF) staining.

**1.11. Wound Healing Assessment**

To evaluate the wound healing capability of the PG, PGC, and PGCA hydrogels, the full-thickness cutaneous defect model was employed. All 25 healthy male Sprague–Dawley rats (200–220 g) were randomly divided into 5 groups (N=4) as follows: a control group without any treatment, Tegaderm^TM^, PG, PGC, and PGCA-treated groups. The rats were anesthetized by IP injection of 0.25 ml of ketamine (50 mg ml^−1^)-xylazine (20 mg ml^−1^) cocktail (6:4 v/v), shaved, and then two rounded full-thickness cutaneous wounds with a diameter of 1.2 cm were created on either backside of each rat. Subsequently, the wounds were fitted with different hydrogel films and covered with Tegaderm ^TM^. The groups with no hydrogel treatment, covered with or without Tegaderm ^TM^ were marked as Tegaderm ^TM^ and control groups, respectively. The wounds were photographed on days 0, 3, 7, 10, and 14 to assess the wound area utilizing ImageJ software. The wound area was determined as follows: Relative wound area$=\frac{A_{n}}{A_{0}}\times100$, where A_0_ and A_n_ are the wound area on day 0 and day n (n=0, 3, 7, 10, and 14), respectively. On day 14, the rats were euthanized and cutaneous tissues of all samples over the wound bed were excised and fixed in 4% formaldehyde overnight. Then the regenerated tissues were embedded in paraffin and cross-sectioned by microtome to 5 µm slices and eventually stained with H&E and Masson’s trichrome for histological evaluation and collagen deposition examination, respectively.

**1.12. Adhesive Properties**

The adhesive property of the PGCA hydrogel was assessed qualitatively. 1 ml of hydrogel (2 h of after preparation) was applied to various biological tissues including heart, lung, muscle, bone, spleen, liver, kidney, and brain from rats, and various substrates including glass, Polytetrafluoroethylene (PTFE), polypropylene (PP), stone, Polydimethylsiloxane (PDMS) sponge, rubber, stainless steel, aluminum, and wood. The adhesiveness of the hydrogel to different substrates was photographed by a digital camera.

**1.13. Wound Closure Efficacy of Hydrogels**

The wound closure capability of the PGCA hydrogel was investigated by a full-thickness rat skin incision model ^7-8^. The Sprague-Dawley rats (Male, 200-230 g) were anesthetized by IP injection of 0.25 ml of ketamine (50 mg ml^−1^)-xylazine (20 mg ml^−1^) cocktail (6:4 v/v). After shaving and sterilization of the surgical area on the skin with 70% ethanol, three full-thickness incisions (2 cm length-3mm depth) were created on the dorsum of the rat. One of the incisions was closed by spreading 200 µl of hydrogel with a needle followed by bringing two edges in contact manually for about 1 min to provide the required adhesion strength to wound closure. The other group was treated with a non-resorbable clinical thread and the last received no treatment as a control group. On the third day after surgery, the animals were euthanized and skin tissue of the wound sites was collected for histological analysis.

**1.14. In Vivo Anti-Adhesion Test**

The anti-adhesion efficacy of the PGCA hydrogel was investigated by a rat model of sidewall defect-cecum abrasion ^9^. In this experiment, 12 male Sprague-Dawley rats (220-250 g) were randomly divided into 2 groups (N=6). Before the experiment, the animals were anesthetized by IP injection of 0.3 ml of ketamine (50 mg l^−1^)-xylazine (20 mg ml^−1^) cocktail (6:4 v/v). After anesthesia, the abdominal skin of the mice was shaved and sterilized with ethanol 70%. Subsequently, the peritoneum was opened by a 5 cm long incision along the midline of the abdomen. The cecum was abraded using a scalpel blade to create damage and hemorrhaging, followed by abrading an area of 1×1 cm^2^ in the internal abdominal wall. For the hydrogel group, a 4×4 cm^2^ PGCA film was applied to cover the abraded abdominal wall and cecum without suturing, while the control group received no treatment. Subsequently, both the muscle and the midline skin incision were sutured with 5-0 silk thread and three rats of each group were euthanized on days 7 and 14 after surgery to check the degree of adhesion. The adhesion scores were assessed in a double-blinded manner according to the standard adhesion scoring system (Table 1) ^10^. Then the tissues of the recovered abdominal wall and adhesion-associated tissues containing the cecum and abdominal wall were collected and fixed by 4% formaldehyde for histological examination after H&E staining.

All the animal care and experimental procedures were performed in accordance with the guideline of laboratory animal center of Zanjan University of Medical Sciences, Zanjan, Iran (ethical code: IR.ZUMS.REC.1399.395). In all in vivo studies, animals were housed in an animal room for one week before starting the experiments, to allow adaptation to the new environment. During the studies, 12 h light/dark daily cycle, an environmental temperature of 21−23 °C, and relative humidity of 50−60% were applied in the animal room.

**Table S1.** Adhesion scoring system.

| Score | Type description |
| --- | --- |
| 0 | No adhesion |
| 1 | One thin filmy adhesion |
| 2 | More than one thin adhesion |
| 3 | Thick adhesion with focal point |
| 4 | Thick adhesion with plantar attachment or more than one thick adhesion with focal point |
| 5 | Very thick vascularized adhesion or more than one plantar adhesion |

**1.15.** **Statistical Analysis**

At least three independent replication for each data set of all experiments were performed and results are reported as mean±standard deviation (SD). Statistical analysis was executed using SPSS 26 software. The statistical significance was estimated by one-way ANOVA with Tukey’s posthoc test.

2. **Results and Discussion**


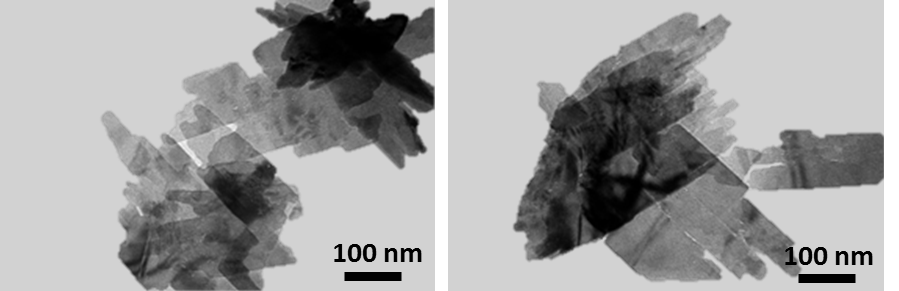


**Figure S1.** TEM images of CuO nanosheets.

**
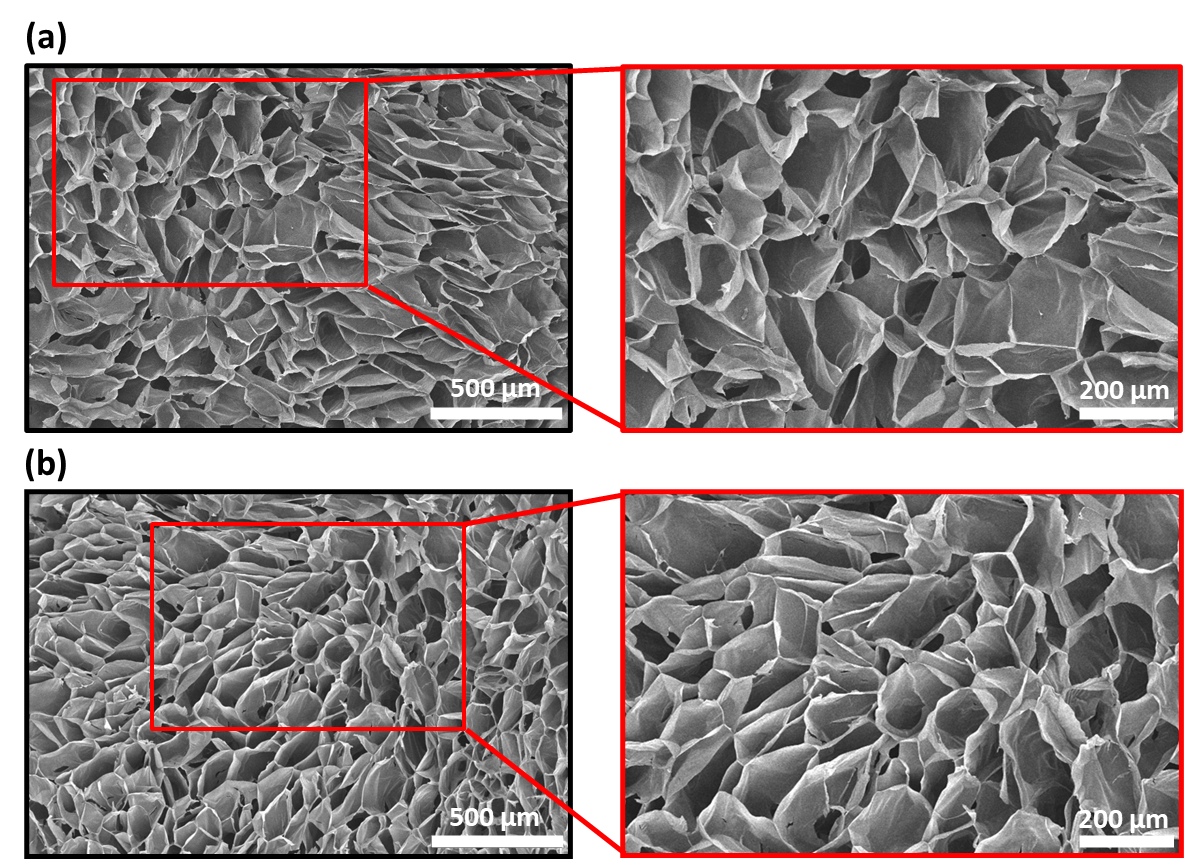
**

**Figure S2.** The SEM images of the hydrogel (a) without and (b) with CuO nanosheets under different magnifications, which showed its high porosity. The addition of the CuO nanosheets to the hydrogel did not change the morphology and pore size of the pores. The porosity measurement study showed porosities of 75.7 ± 5.2% and 73.9 ± 4.1% for hydrogels without and with CuO nanosheets, respectively.

**Yield and degradation of hydrogels**

The yields percentages of all three hydrogels were studied to evaluate the crosslinking reaction efficiency and possible interference of CuO nanosheets and allantoin in gel formation. All three hydrogels had more than 95% yield without any significant difference (Figure S3a), which confirmed the CuO nanosheets and allantoin would not alter the yield of the PG hydrogel.

The degradation rate of the PG, PGC, and PGCA hydrogels was carried out in PBS (pH 7.4) at 37 ˚C (Figure S3b). The results showed that the hydrogels degrade over time and PG and PGCA hydrogels showed a higher degradation rate due to the probable interference of CuO nanosheets and allantoin with the crosslinker during the hydrogel formation, which is in accordance with the swelling ratio.

**
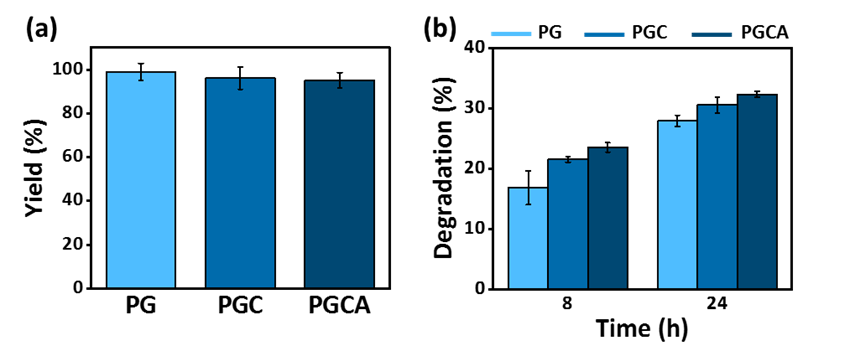
**

**Figure S3.** (a) The yield and (b) degradation of PG, PGC, and PGCA hydrogels.

**ATR-FTIR Analysis**

The chemical structure of pure materials and PG, PGC, and PGCA hydrogels were evaluated using ATR-FTIR spectroscopic analysis (Figure S4). PMVE-MA, formed by PMVE-MAH hydrolysis, showed the typical C=O stretching band at 1862 cm^−1^ and 1780 cm^−1^, asymmetrical stretching vibrations of –COO– at 1705 cm^-1^, and O-H bending band at about 3477 cm^−1^. Moreover, the C–H stretching vibrations was observed at 2923 cm^-1^.^11^ In the gelatin spectrum, a broad band was observed at 3446 cm^−1^ arising from the N-H stretching of amide A and free O-H. The C-H stretching of amide B, C=O stretching of the amide I, and N-H deformation of the amide II were detected at 2923, 1641, and 1538 cm^−1^, respectively. The vibration of proline side chains was also found at 1332 cm^−1^.

In the PG spectrum, the more right shift in wavenumbers of the carbonyl group (1729 cm^−1^) in comparison to PMVE-MA (1780 cm^−1^) confirmed the successful ring-opening of PMVE-MA and esterification reaction between the acid groups of PMVE-MA with the hydroxyl groups of opened PEGDGE. In the same way, after crosslinking between NH_2_ of gelatin and PEGDGE, the N-H vibration was shifted to a lower wavelength at 3436 cm^−1^ with a lower frequency while it was observed for gelatin at 3473 cm^−1^. The characteristic stretching of C-O-C of PEGDGE at 1095 and 950 cm^−1^ and also C-H bending vibration were found at 1349 and 840 cm^−1^ in all three hydrogels. Moreover, the C-H stretching band of methylene in PEGDGE and gelatin was merged and appeared as an intense band at about 2867 cm^−1^ in the hydrogels ^12^.

The emergence of the band at about 600 cm^−1^ confirmed the presence of CuO nanosheets in the PGC and PGCA hydrogels in comparison to PG. Most of the associated absorption bands of allantoin was also observed in PGCA hydrogel in comparison to PG and PGC. In the spectrum of PGCA hydrogel, the allantoin-related double stretching vibration bands of NH_2_ were observed at 3342 and 3436 cm^−1^ among the wide OH and NH band at about 3400 cm^−1^ in comparison to PGC hydrogel. Moreover, the C=O stretching characteristic of allantoin at 1778, 1712, and 1650 cm^−1^ was found in the PGCA hydrogel. All the results confirmed the crosslinking of polymers and hydrogel formation, as well as the incorporation of CuO nanosheets and allantoin into the hydrogel network.

**Figure S4.** ATR-FTIR of pure materials and PG, PGC, and PGCA hydrogels.

**XRD Analysis**

The structure of the pure materials and PG, PGC, and PGCA hydrogels was verified using XRD analysis (Figure S5). The XRD pattern of PMVE-MA showed a broad peak at 2θ**~**16.5°, suggesting the amorphous structure. The broad peak at 2θ**~**19 ° was assigned to the triple-helical crystalline structure of gelatin, indicating the partially crystalline structure ^13^. The diffraction peak of gelatin in PG hydrogel was shifted to 21 °θ due to the crosslinking reaction. The high degree crystalline nature of CuO nanosheets was confirmed by sharp peaks at 2θ of 32.7, 35.9, 39.1, 46.5, 49.2, 53.8, 58.5, 61.9, 66.6, 68.3, 72.9 and 75.3 ° corresponded to (1 1 0), (-1 1 1, 0 0 2), (1 1 1, 2 0 0), (-1 1 2), (-2 0 2), (0 2 0), (2 0 2), (-1 1 3), (-3 1 1), (1 1 3), (3 1 1), and (0 0 4), respectively ^14^. However, no defined peaks of CuO nanosheets were observed in PGC and PGCA hydrogels due to the very low amount of CuO nanosheets in the hydrogel network. XRD spectrum of allantoin displayed many sharp diffraction peaks, confirming the crystalline structure of allantoin. The two characteristic peaks of allantoin in the PGCA hydrogel were appeared at 2θ**~**32 ° with a very low intensity due to the small amount of allantoin in the hydrogel network. Moreover, the assigned peaks of allantoin in the PGCA hydrogel were shifted to the higher 2θ in comparison to pure allantoin owning to the disturbing effect of hydrogel to the crystallinity of allantoin.

**Figure S5.** XRD patterns of pure materials and PG, PGC, and PGCA hydrogels.

**TGA and DTG Analysis**

TGA and DTG curves of pure materials and PG, PGC, and PGCA hydrogels are demonstrated in Figure S6a and S6b, respectively. As shown in TGA thermogram of PMVE-MA, the weight loss at temperature of about 160 °C was due to the dehydration of the carboxyl groups of the PMVE-MA and the anhydride carboxylic acid formation. The main weight loss step started at 260 °C corresponded to the destruction of the polymer backbone ^15^. The main weight loss of gelatin was started at 250 °C and the total weight loss was 80.8% at 800 °C. PEGDGE showed the main step of weight loss at 160–370 °C with T_50%_ of 305 °C, and almost no residue was maintained at the temperature of 800 °C. TGA curve of CuO nanosheets showed a small amount of weight loss at~200 °C due to the evaporation of water. The second mass loss at 750 °C corresponded to the pyrolysis and calcination of metal oxide ^16^. The total weight loss of CuO nanosheets was less than 10% up to 800 °C. The thermogram of allantoin showed three steps of weight loss and 7.5 % char was left at 800 °C. TGA curves of PG, PGC, and PGCA hydrogels showed two main weight loss steps. The first step was regarded to the evaporation of crystalline and absorbed water on the surface of materials and the second large weight loss was related to the material decomposition of hydrogels which continued up to 430 °C. The residue at 800 °C was 9.1%, 9.9%, and 10.2% for PG, PGC, and PGCA, respectively. The higher ash of PGC and PGCA hydrogels in comparison to PG is attributed to the presence of CuO nanosheets. Figure S6c summarizes the temperatures at 10% and 50% weight loss and also the residual at 800 °C for different materials.

DTG curves of the hydrogels showed that the maximum decomposition rate was at 390 °C with higher degradation speed in comparison to initial components. Also, the main decomposition occurred at a higher temperature in comparison to the pure components, confirming the improved thermal stability of the hydrogels through the crosslinking reaction.


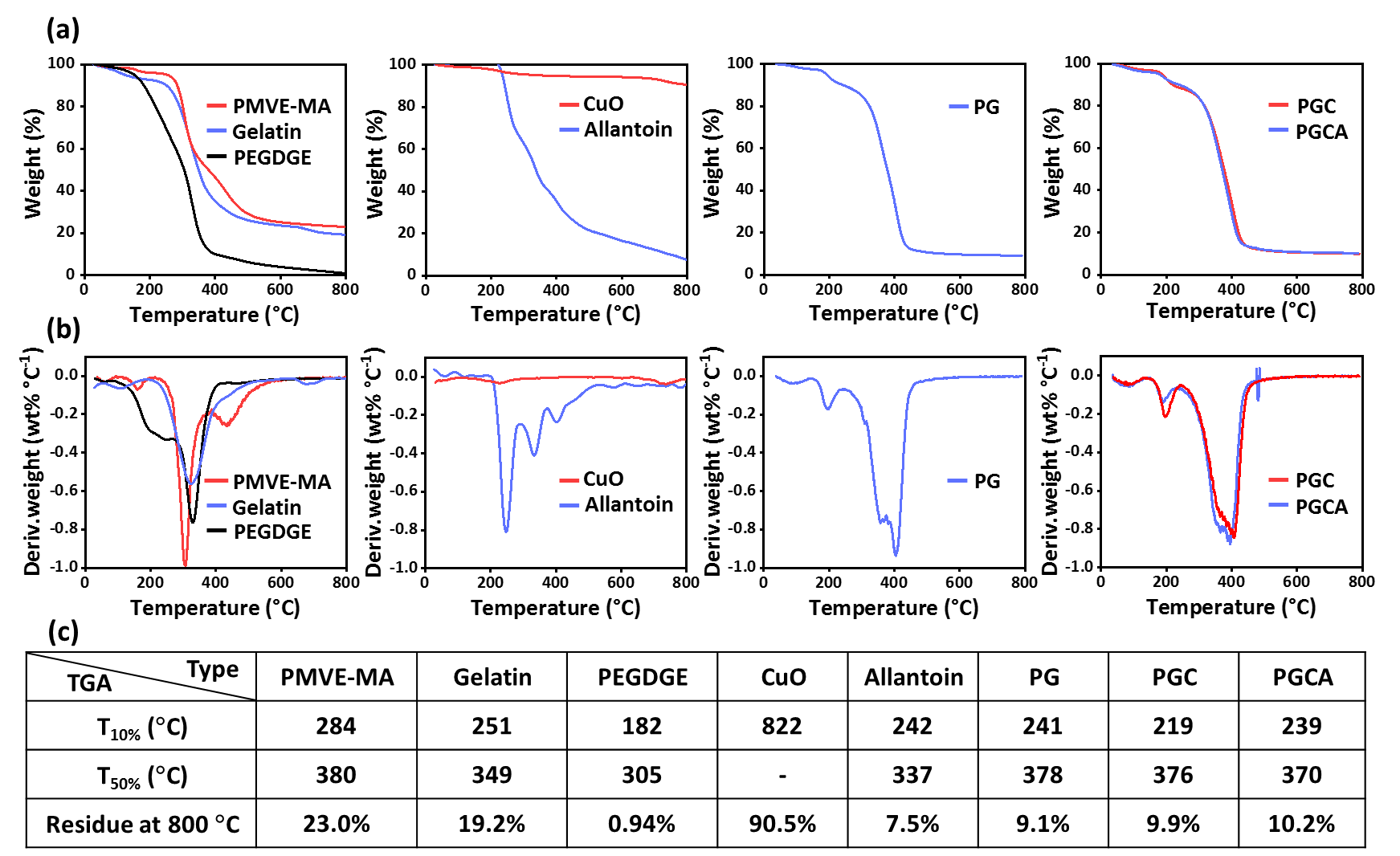


**Figure S6**. The (a) TGA and (b) DTG thermograms of pure materials, PG, PGC, and PGCA hydrogels. (c) The temperature at 10% (T 10%) and 50% (T 50%) weight loss, and the residual amount at 800 °C for different materials reclaimed from TGA.

To show the injectability and shape maintaining ability of the hydrogel, “PN” was written by PGCA hydrogel 2 h after preparation. As shown in Figure S7a, the hydrogel could be injected out from a 21-guage needle without any clogging and the structure of the hydrogel was maintained successfully.

**
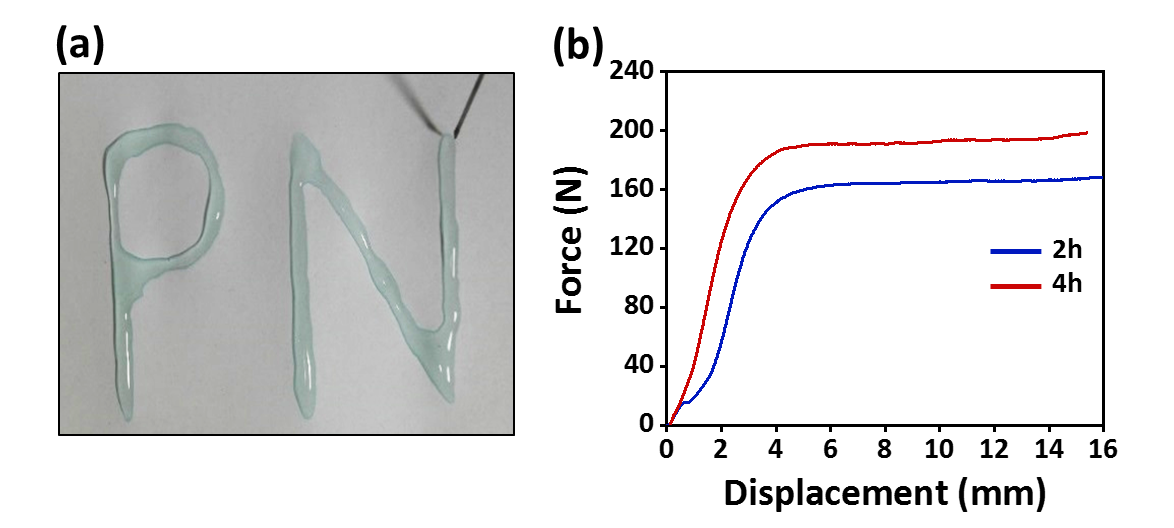
**

**Figure S7.** (a) The image of PGCA hydrogel extrusion through a 21-guage needle 2 h after preparation. (b) Injectability force versus displacement curve for PGCA hydrogel to be injected through a 10-ml syringe with a 21-guage needle 2 and 4h after preparation at room temperature.

**Mechanical Properties**

**
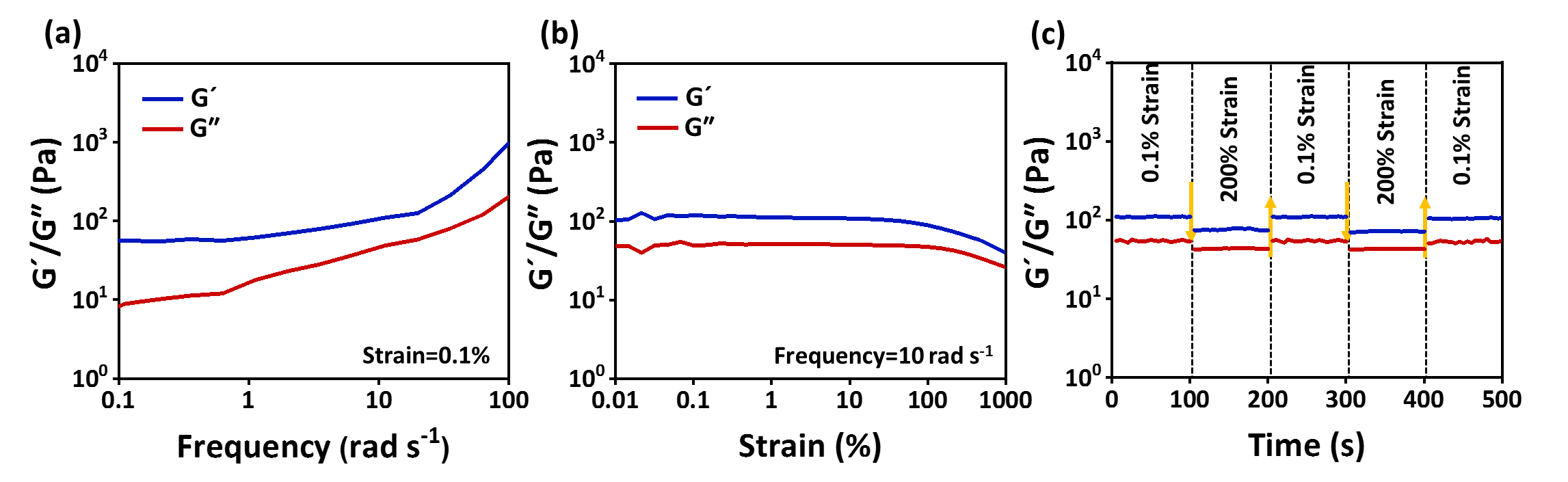
**

**Figure S8.** (a) Frequency sweep at strain of 0.1%, (b) strain amplitude sweep at frequency of 10 rad s^-1^, and (c) alternate step strain test of PG hydrogel 48 h after preparation.


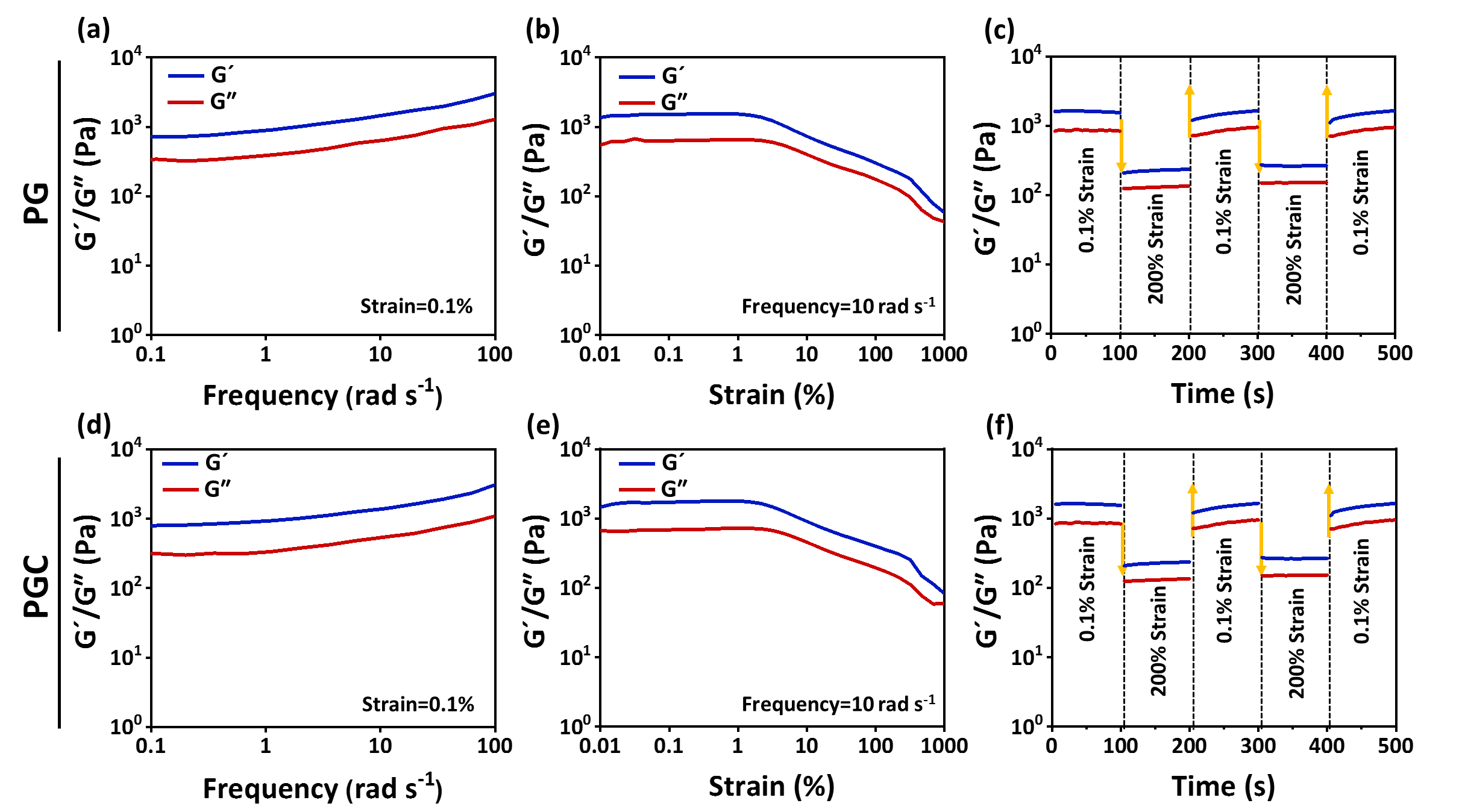


**Figure S9.** (a,d) Frequency sweep test at strain of 0.1%, (b,e) strain amplitude sweep test at frequency of 10 rad s^-1^, and (c,f) alternate step strain test of PG and PGC hydrogels, respectively, 10 days after preparation.


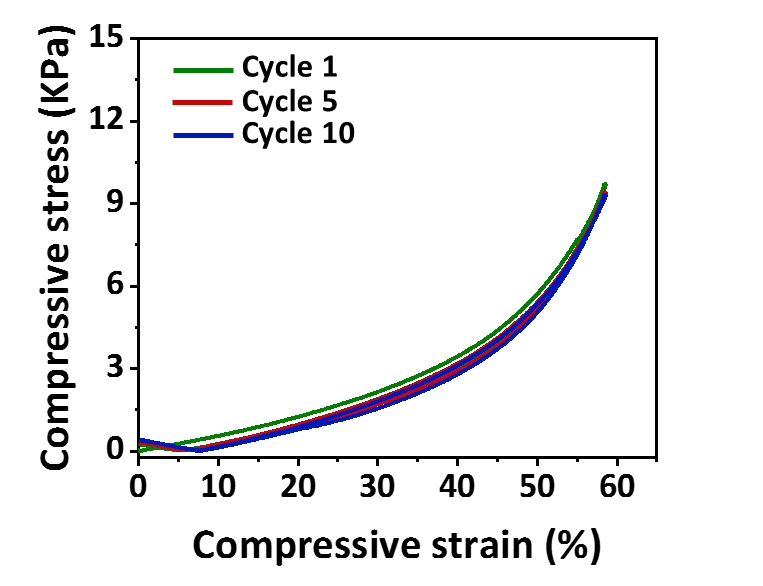


**Figure S10.** Cyclic compressive loading-unloading test of the PGCA hydrogel at strains of 0-60% 10 days after preparation.


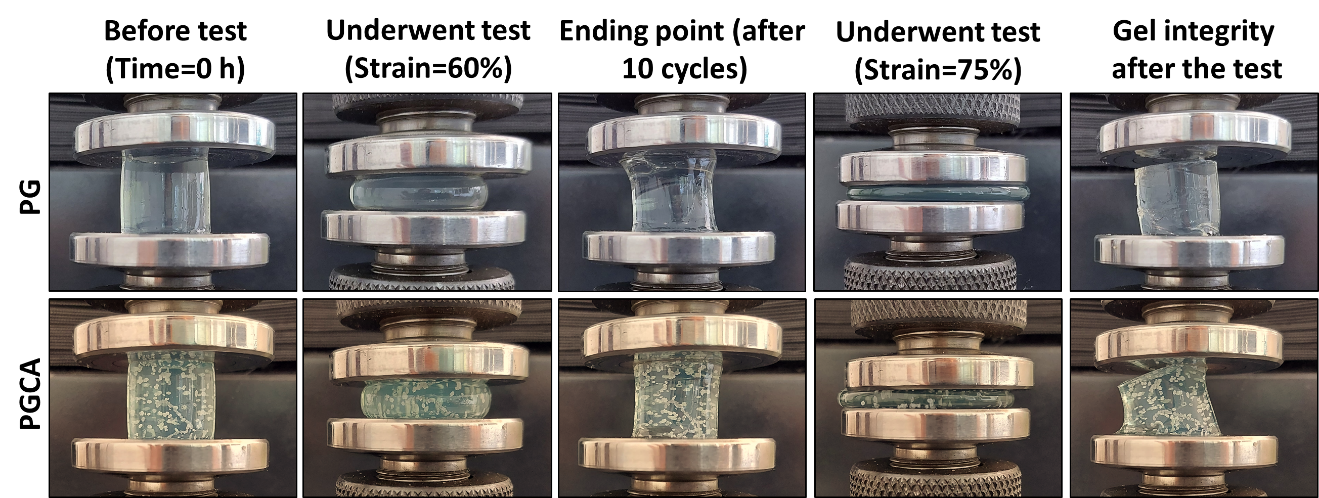


**Figure S11.** Evaluation of appearance characteristics of the PG hydrogel before, during, and after the compression test.

**Moldability of the Hydrogel**

Moldability of the hydrogel is attributed to the plasticizing effect of the PEGDGE, which allows the hydrogel to be utilized as an ideal wound dressing in the curvilinear area of the human body like the ankle and wrist, while resisting external forces.


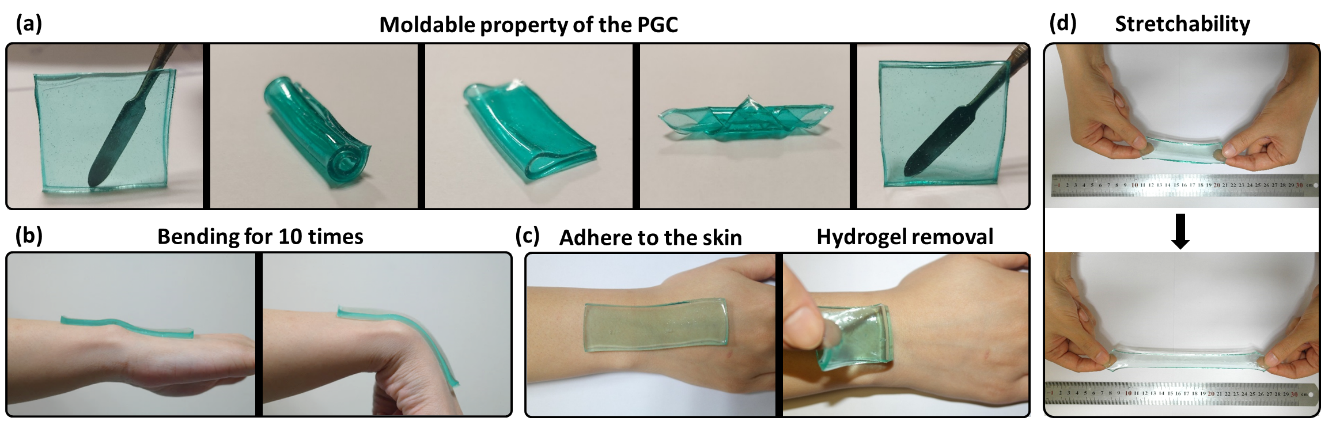


**Figure S12.** Photographs representing (a) flexibility and backing to the original shape, (b) ability to bend, (c) adhesion and removal, and (d) stretchability of the film-formed PGC hydrogel.

**Biocompatibility of the Hydrogel**


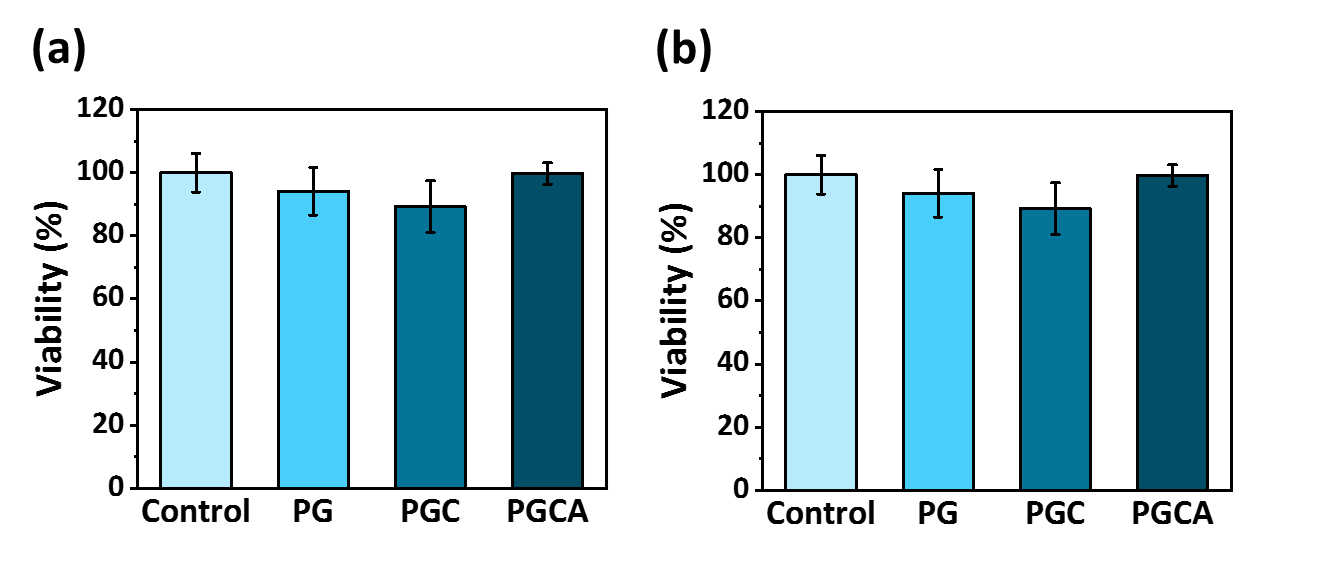


**Figure S13.** The viability of (a) NIH/3T3 and (b) HDFa normal fibroblasts after treatment with PG, PGC, and PGCA hydrogels at 37 °C for 24 h. PGCA hydrogel demonstrated high safety with a cell viability of higher than 94% for both cell lines after 24 h.


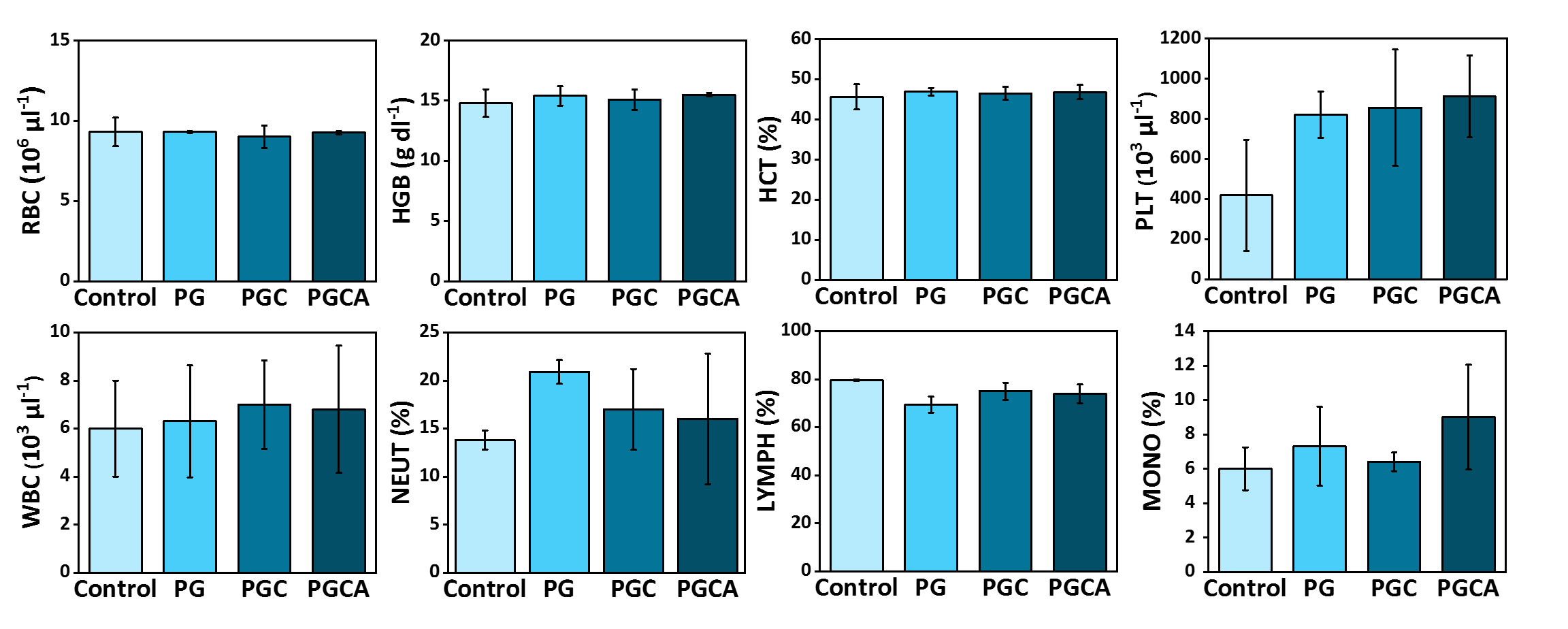


**Figure S14.** In vivo toxicity evaluation of PG, PGC, and PGCA hydrogels. Hematological factors for different treated groups after 14 days. Data are reported as mean±SD (N=4). The statistical analysis was performed by One-way ANOVA (*p<0.05 *vs*. control group).


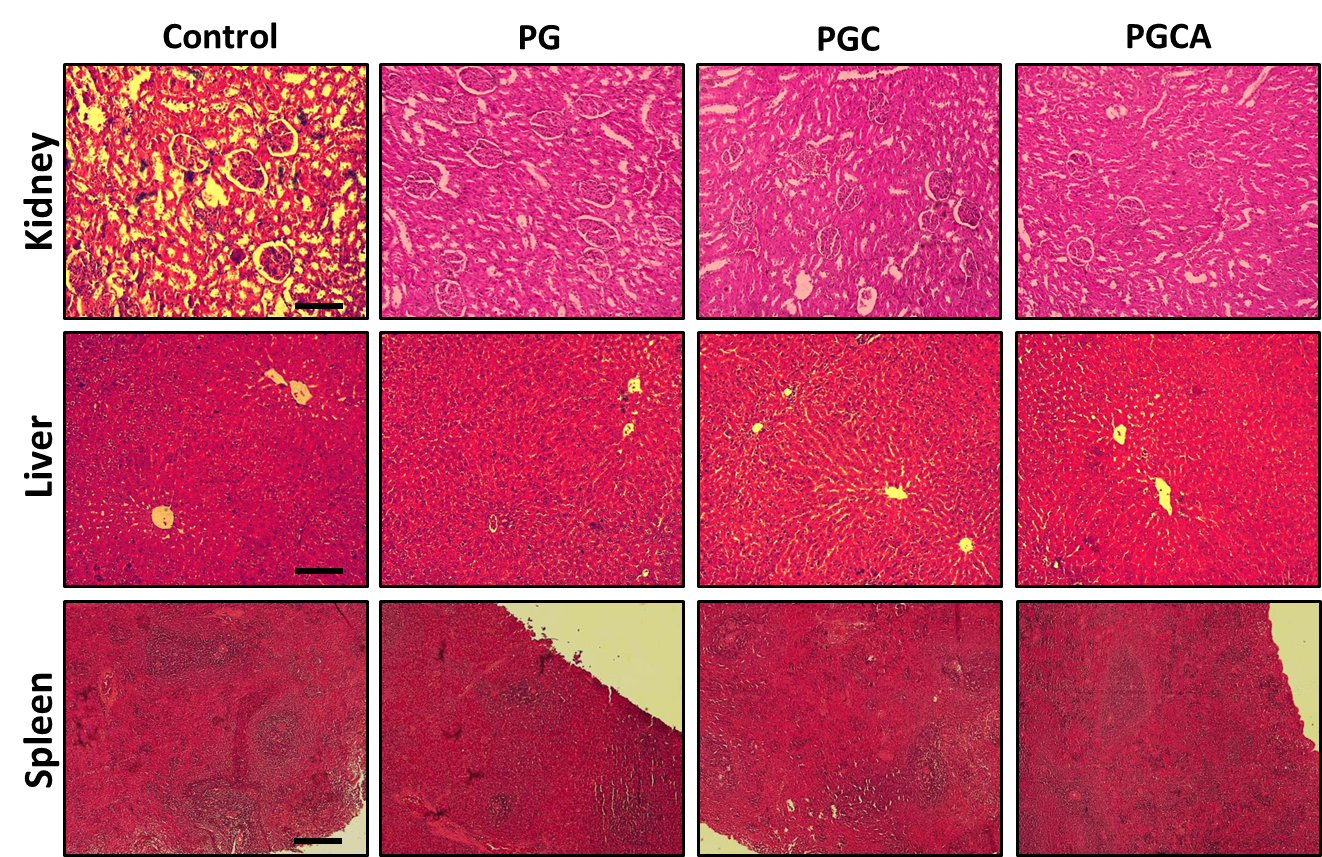


**Figure S15.** In vivo toxicity evaluation of PG, PGC, and PGCA hydrogels. H&E stained kidney, liver, and spleen tissues 14 days after subcutaneous injection of the hydrogels in rats. Scale bar=200 µm.

**Histological Studies of the Infected Tissue**


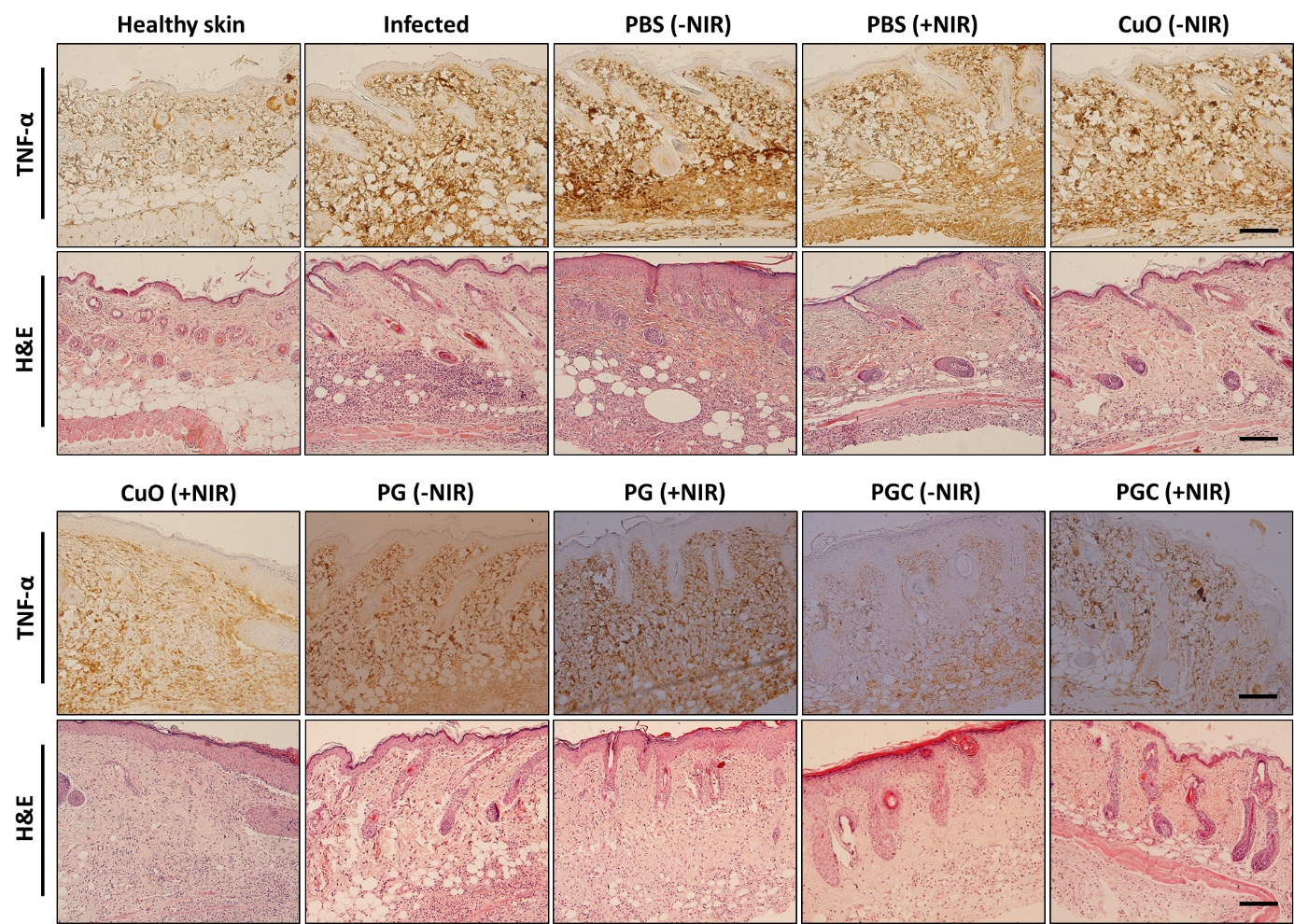


**Figure S16.** H&E staining and immunohistochemical study of TNF-α expression in the infected skin tissues of different groups in the murine abscess model after 13 days. Scale bar=200 µm.

**Histological studies of the Tumor Tissue**


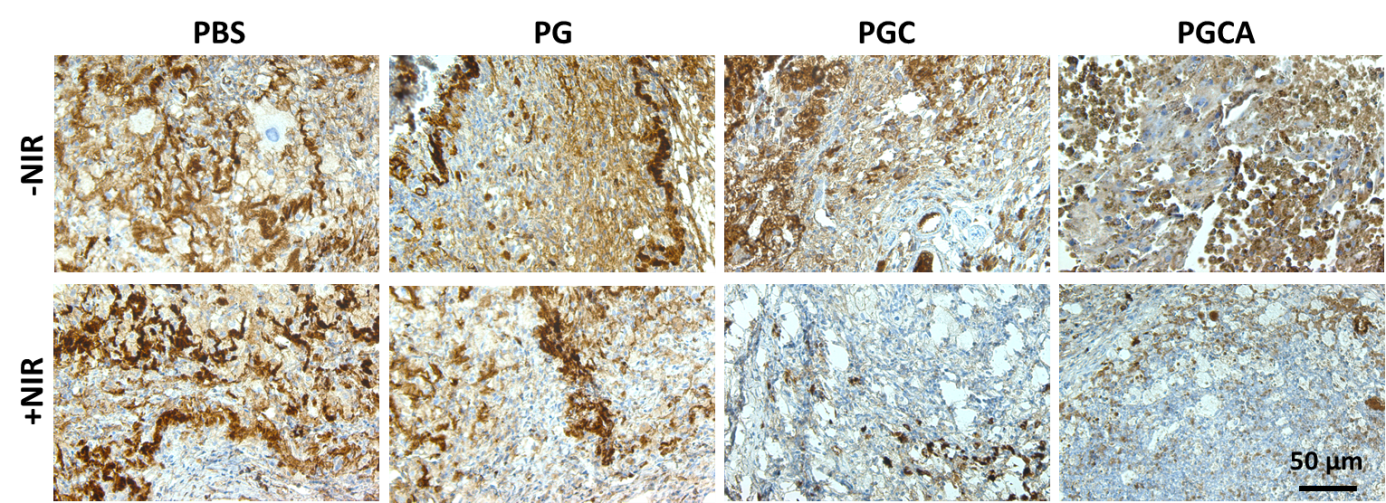


**Figure S17.** VEGF staining of the tumor tissues 15 days after treatment. Scale bar=50 µm.


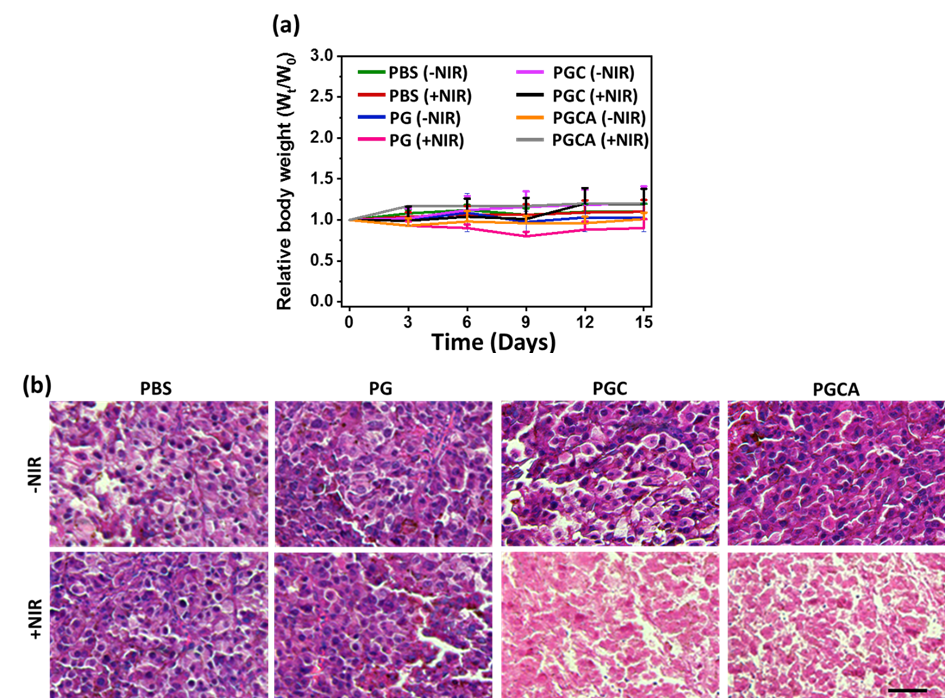


**Figure S18.** (a) The relative change of body weight in PBS (±NIR), PG (±NIR), PGC (±NIR), and PGCA (±NIR)-treated groups during 15 days screening. (b) H&E staining of the tumor tissues 15 days after treatment. Scale bar=20 µm.


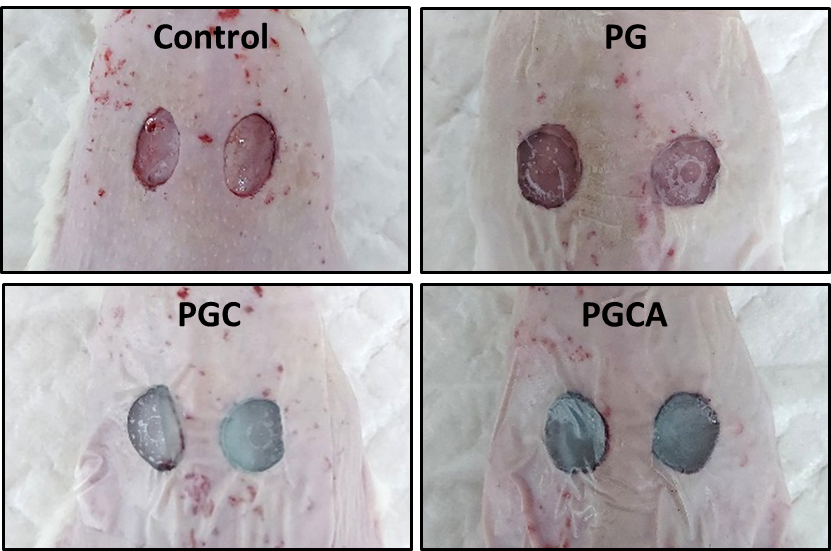


**Figure S19.** The images of establishing a full-thickness cutaneous wound model in rats followed by treatment with PG, PGC, and PGCA films as a wound dressing, which was covered by Tegaderm^TM^.


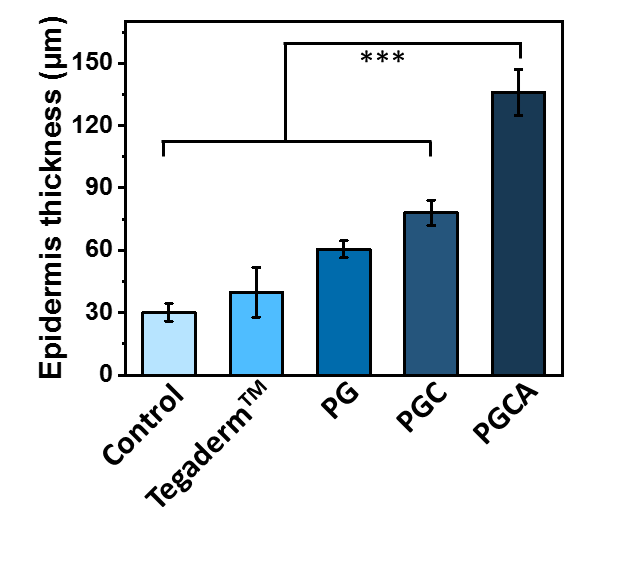


**Figure S20**. Epidermis thickness of the control, Tegaderm^TM^, PG, PGC, and PGCA-treated groups in the full-thickness cutaneous wound model in rats at day 14 obtained from H&E-stained tissues. Data are presented as mean±SD (N=3; ***p<0.001).

**Figure S21.** Drug release graph showing cumulative drug release (%) from the final hydrogel during 72 h. Data are presented as mean±SD (N=3).


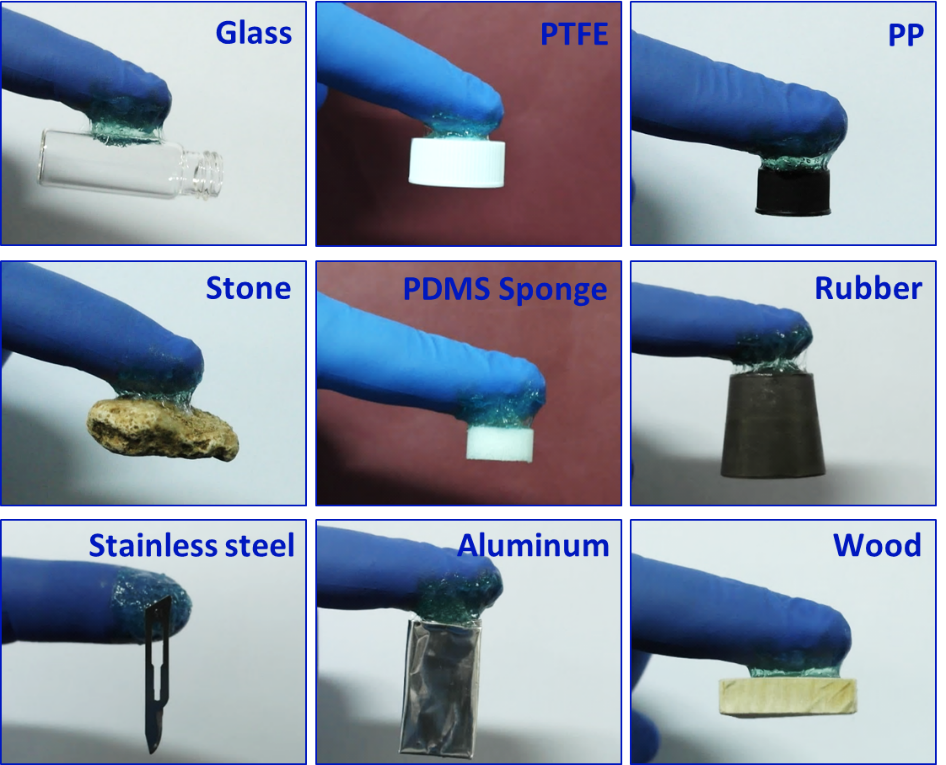


**Figure S22**. Adhesion exhibition of the PGCA hydrogel to various substrates, including glass, PTFE, PP, stone, PDMS sponge, rubber, stainless steel, aluminum, and wood.


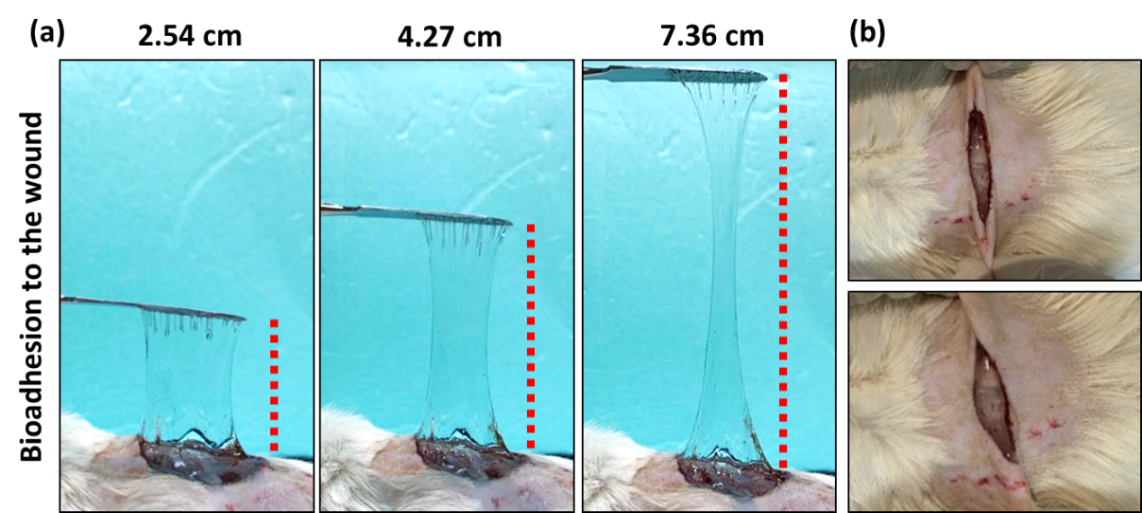


**Figure S23.** (a) Adhesion exhibition of the PGCA hydrogel to the wound site and (b) cohesive adhesion of PGCA hydrogel to the wound site after twisting the skin of rat.

**References:**

1. Gorgizadeh, M.; Azarpira, N.; Veis, R. D.; Sattarahmady, N. J. C.; Biointerfaces, S. B., Repression of melanoma tumor in vitro and in vivo by photothermal effect of carbon xerogel nanoparticles. *Colloids Surf., B* **2019,** *176*, 449-455.

2. Qu, J.; Zhao, X.; Liang, Y.; Zhang, T.; Ma, P. X.; Guo, B. J. B., Antibacterial adhesive injectable hydrogels with rapid self-healing, extensibility and compressibility as wound dressing for joints skin wound healing. *Biomaterials* **2018,** *183*, 185-199.

3. Lv, Q.; Wu, M.; Shen, Y. J. C.; Physicochemical, S. A.; Aspects, E., Enhanced swelling ratio and water retention capacity for novel super-absorbent hydrogel. *Colloids Surf., A* **2019,** *583*, 123972.

4. Robinson, T. E.; Hughes, E. A.; Eisenstein, N. M.; Grover, L. M.; Cox, S. C., The quantification of injectability by mechanical testing. *JoVE (Journal of Visualized Experiments)* **2020,** (159), e61417.

5. Wu, F.; Chen, L.; Yue, L.; Wang, K.; Cheng, K.; Chen, J.; Luo, X.; Zhang, T., Small-molecule porphyrin-based organic nanoparticles with remarkable photothermal conversion efficiency for in vivo photoacoustic imaging and photothermal therapy. *ACS Appl. Mater. Interfaces* **2019,** *11* (24), 21408-21416.

6. Zhao, X.; Guo, B.; Wu, H.; Liang, Y.; Ma, P. X. J. N. c., Injectable antibacterial conductive nanocomposite cryogels with rapid shape recovery for noncompressible hemorrhage and wound healing. *Nat. Commun.* **2018,** *9* (1), 1-17.

7. Meddahi‐Pellé, A.; Legrand, A.; Marcellan, A.; Louedec, L.; Letourneur, D.; Leibler, L. J. A. C. I. E., Organ repair, hemostasis, and in vivo bonding of medical devices by aqueous solutions of nanoparticles.  *Angew. Chem., Int. Ed.* **2014,** *53* (25), 6369-6373.

8. Gao, L.; Zhou, Y.; Peng, J.; Xu, C.; Xu, Q.; Xing, M.; Chang, J. J. N. A. M., A novel dual-adhesive and bioactive hydrogel activated by bioglass for wound healing. *NPG Asia Mater.* **2019,** *11* (1), 1-11.

9. Zhang, E.; Li, J.; Zhou, Y.; Che, P.; Ren, B.; Qin, Z.; Ma, L.; Cui, J.; Sun, H.; Yao, F. J. A. b., Biodegradable and injectable thermoreversible xyloglucan based hydrogel for prevention of postoperative adhesion. *Acta Biomater.* **2017,** *55*, 420-433.

10. Zhang, E.; Guo, Q.; Ji, F.; Tian, X.; Cui, J.; Song, Y.; Sun, H.; Li, J.; Yao, F. J. A. b., Thermoresponsive polysaccharide-based composite hydrogel with antibacterial and healing-promoting activities for preventing recurrent adhesion after adhesiolysis. *Acta Biomater.* **2018,** *74*, 439-453.

11. Kerdsakundee, N.; Li, W.; Martins, J. P.; Liu, Z.; Zhang, F.; Kemell, M.; Correia, A.; Ding, Y.; Airavaara, M.; Hirvonen, J. J. A. h. m., Multifunctional nanotube–mucoadhesive poly (methyl vinyl ether‐co‐maleic acid)@ hydroxypropyl methylcellulose acetate succinate composite for site‐specific oral drug delivery.  *Adv. Healthcare Mater.* **2017,** *6* (20), 1700629.

12. Teng, X.; Xu, H.; Song, W.; Shi, J.; Xin, J.; Hiscox, W. C.; Zhang, J. J. A. o., Preparation and properties of hydrogels based on PEGylated lignosulfonate amine. *ACS omega* **2017,** *2* (1), 251-259.

13. Das, M. P.; Suguna, P.; Prasad, K.; Vijaylakshmi, J.; Renuka, M., Extraction and characterization of gelatin: a functional biopolymer. *Int. J. Pharm. Pharm. Sci* **2017,** *9* (9), 239.

14. Zhang, H.; Wang, K.; Wang, L.; Xie, H.; Yu, W. J. S. E., Mesoporous CuO with full spectrum absorption for photothermal conversion in direct absorption solar collectors. *Sol. Energy* **2020,** *201*, 628-637.

15. Torres-Figueroa, A. V.; Pérez-Martínez, C. J.; Encinas, J. C.; Burruel-Ibarra, S.; Silvas-García, M. I.; García Alegría, A. M.; del Castillo-Castro, T., Thermosensitive Bioadhesive Hydrogels Based on Poly (N-isopropylacrilamide) and Poly (methyl vinyl ether-alt-maleic anhydride) for the Controlled Release of Metronidazole in the Vaginal Environment. *Pharmaceutics* **2021,** *13* (8), 1284.

16. Rami, J.; Patel, C.; Patel, C.; Patel, M., Thermogravimetric analysis (TGA) of some synthesized metal oxide nanoparticles. *Materials Today: Proceedings* **2021,** *43*, 655-659.
